# The global emission mitigation potential of avoiding waste and product lifespan extension by Chinese households

**DOI:** 10.1016/j.heliyon.2024.e24322

**Published:** 2024-01-23

**Authors:** Bingqian Yan, Erik Dietzenbacher, Bart Los

**Affiliations:** a National Academy of Economic Strategy, Chinese Academy of Social Sciences, Beijing, 100006, China; b Faculty of Economics and Business, University of Groningen, PO Box 800, 9700 AV, Groningen, the Netherlands

**Keywords:** Global carbon footprint, Scenario analysis, Low-carbon behavior, Lifestyle change, Input-output analysis, Engel curves

## Abstract

This paper examines the emission mitigation potential of Chinese households’ low-carbon behavior by 2030 through a global carbon footprint scenario analysis. The emission reduction effect is estimated by comparing the projected global emissions in 2030 in a lifestyle emulation scenario and a low-carbon scenario, in which Chinese households adopt low-carbon consumption behaviors. Lifestyle emulation is modeled based on what we call “world Engel curves”, which describe how the expenditure share of a certain consumption good depends on the total per capita expenditures for household consumption (which depends on income). By including a dynamic link between household lifestyle changes and GDP, we then obtain the emission projections under different scenarios in 2030, based on the historical data for 49 countries from 1995 to 2011 from EXIOBASE. Our results show that adopting a mild low-carbon lifestyle by households helps only little in terms of reducing GHG emissions. Reducing avoidable waste and expanding the lifetime of products are not enough to help meeting the 2 °C goal. More drastic changes are required.

## 1 Introduction

Increasing greenhouse gas (GHG) emissions are recognized as the major cause of global warming and climate change [1]. As the largest emitter country,^1^ China faces much pressure to reduce emissions. The development of clean production technology (such as Carbon Capture and Storage) and renewable energy can help reduce emissions in the future [1]. At the same time, however, increasing consumption will exert the opposite force and is expected to be the core driver of growing emissions in China in the coming years.

Although China is the second largest economy in 2020, the GDP per capita is still modest if viewed from a global perspective, at around 1/6 of that of the US according to World Bank data.^2^ It is clear that with economic growth and income increase, more and more people can afford products they could not buy before. The demand of citizens will change from basic needs to an increasing variety of goods and services. The lifestyle in industrialized countries will be imitated by people in emerging countries. For instance, the ownership of air conditioners in China has increased four times from 20 sets per 100 households in 1998 to 118 in 2020. The ownership of mobile phone even increased from 3 phones per 100 households in 1998 to 254 in 2020 in China [2]. As Duchin and Lange (1994) already pointed out, the emulation of lifestyle in developed countries by people in developing countries will exert a high pressure on the environment [3]. Increasing consumption in urbanizing China has been identified as an important driver of growing household carbon footprints over the past 20 years [[4], [5], [6]]. Between 2007 and 2012 the household footprint increased by 19%, with 75% of the increase due to increased consumption of the urban middle class and the rich [6]. Differently, if households adopt low-carbon behaviors when getting rich, global emissions would reduce consequently. Therefore, it is important for household to change behaviors dramatically to contributing to emission mitigation.

This suggests that a transformation of Chinese lifestyles away from the current path of adopting more carbon-intensive consumption patterns is needed to achieve carbon neutrality. On the one hand, citizens have a higher willingness to take measures to achieve cleaner air [7], since the severe air pollution nowadays in China leads to many health problems [8]. On the other hand, in January 2022, the National Development and Reform Commission in China released its *Implementation Plan for Promoting Green Consumption* [9]. The plan states that “[b]y 2030, green consumption patterns will become the conscious choice of the public, green and low-carbon products will become mainstream, a green and low-carbon consumption model in key areas will be basically formed, and a sound green consumption system, policy system, and institutional mechanisms will be formed”.^3^ This leads automatically to the following three research questions of this paper. (1) How will the Chinese household consumption bundle change when China develops further under two different scenarios? In the first of these scenarios, China (and all other countries in the world) emulates the lifestyle of developed countries, whereas China chooses to adopt a low-carbon lifestyle in the second scenario. (2) By how much can greenhouse gas (GHG) emissions be reduced by 2030 if Chinese households adopt a low-carbon behavior and what can the change in the global carbon footprint of Chinese household consumption be in that case? (3) Which consumption products have the highest potential to reduce emissions, and what are the implications for consumers to effectively reduce emissions? To answer these questions, the study will conduct a global footprint scenario analysis.

Many scenario analyses have studied the environmental effect of economic growth and lifestyle change [3,[11], [12], [13]]. Few of them, however, compare the different choices (adopting a low-carbon lifestyle vs. lifestyle emulation) that Chinese households have when they get rich.^4^ This paper applies a global input-output model to estimate the environmental impact of future lifestyle changes. We focus on the carbon footprint of households which gives us the global emissions embodied in the household consumption bundle. Our scenario analysis differs from previous analyses in three aspects.

First, lifestyle emulation is modeled based on what we call “world Engel curves”, which describe how the expenditure share of a certain consumption good depends on the total expenditures for household consumption (which depends on income). The curves are estimated for 200 products with annual data for 49 countries in the period 1995–2011. Since the household consumption shares for emissions-intensive products (such as products for mobility and shelter) are higher in industrialized countries, lifestyle emulation will lead to an emission increase. Second, we include a dynamic link between household lifestyle change and GDP. Specifically, household consumption in the current year determines current-year’s GDP, which determines household consumption in the next year. Then, the economic and environmental impacts of lifestyle changes are calculated up to 2030 by running the dynamic model multiple times, with different sets of parameter values. Third, because of the dynamic links, the model incorporates induced effects, which affect other final demands, such as investment demand. That is, Chinese final demands other than household consumption and final demands in other countries are also affected by consumption behavior of Chinese households. If Chinese households change their consumption pattern (e.g. because they adopt a low-carbon lifestyle), China’s GDP will be affected. This means that next year, not only Chinese household consumption but also the other final demands will change. In the same vein, if Chinese households change their consumption pattern, imports will change and, thus, GDP of other countries will be affected and, consequentially, final demand in these countries in the next year.

Specifically, the scenario model is built with the environmentally extended global input-output tables from 1995 to 2011 from EXIOBASE version 3 with high sector detail. The dataset includes 44 countries and 5 rest-of-world regions, and each country has 200 products [14]. We consider two types of scenario. The baseline scenario (BL) where all countries emulate their lifestyles according to the world Engle curves and one (LC) where (only) Chinese households adopt a low-carbon lifestyle. In the low-carbon scenario, Chinese households adopt the dematerialized lifestyle behavior and avoid unnecessary wastes,^5^ as documented in the literature [15,16]. As a robustness check we also apply these two scenarios to the case where Chinese final demands and intermediate inputs are assumed to become more dependent on domestic suppliers and less on foreign suppliers. This assumption is made on the basis of the revealed historical trend in global markets and China’s recent policy on promoting domestic consumption [17,18].

The rest of the paper is organized as follows. Section 2 reviews the related literature. Section 3 describes the data and the scenario model used in this paper. The results and discussions are given in Section 4. Section 5 concludes.

## 2 Literature review

This study is related to two strands of literature: the first is about footprint analysis and the second is about scenario analysis of consumption changes. For measuring the footprint of household consumption, the existing literature distinguishes two types of methods: life cycle assessments and input-output analyses. Life cycle assessments are commonly applied to assess the environmental impact of specific products and services at the micro level, for example a single product. They account for the emissions or resources used along the product’s life cycle from production to distribution and to disposal [22,23]. In contrast, to evaluate the environmental effect along the full life cycles of all products, environmentally extended input-output analysis is widely used [24,25]. This includes all supply chain effects avoiding the need to define a boundary for analysis [26]. Multi-regional input-output (MRIO) tables describe the whole global production structure and are thus used in this type of analysis. Because we are interested in the footprints of the full bundles of consumption goods, environmentally extended MRIO analysis is also used in this paper.

In the literature, several studies have analyzed the carbon footprint of household consumption to identify possible sustainable consumption patterns by using environmentally extended MRIO analysis [6,[27], [28], [29]]. For example, Hertwich and Peters (2009) found that 72% of global GHG emissions in 2001 are related to household consumption, 10% to governmental final expenditure, and 18% to investments. Looking at product categories, food accounted for 20% of GHG emissions, residence for 19%, and mobility for 17% [27]. For China, Sun et al. (2021) found that housing contributed 45% to the footprint of households, transportation 20%, and food also 20% [29].

This paper is also closely related to studies that use a scenario model to analyze future consumption pattern changes and formulate a sustainable consumption policy. Duchin and Lange (1994) used an input-output model of the world economy to evaluate the scenario described in the Brundtland Report [3]. The model divided the world into sixteen regions, which are linked by trade in goods and services, and by flows of capital over the period 1980–2020. The results showed that the positive future prospects (of increased fuel-efficiency and other technological advances) as described in the Brundtland Report, will not achieve sustainable development in the long term. They suggested bolder technological and social changes to meet the sustainable development goal. Focusing on a specific country, Hubacek et al. (2009) took predictions for the urbanization and lifestyle changes in China by 2020, and applied a single country input-output analysis to calculate the implications for the emission and water footprints [13]. Weber and Perrels (2000) considered lifestyle factors in relation to future energy demand in West Germany, France, and the Netherlands in 2010 [30]. They accounted for household characteristics in deciding on the energy services (e.g., space heating, electronic use, and demand for private cars). They found that a scenario where consumers attached more attention to the environmental quality of products achieved the largest reduction in household energy consumption. Recently, De Koning et al. (2016) used supply-use tables to study whether predicted technological developments can reduce CO2 emissions in 2050 to a level that is consistent with the goal of a maximum temperature rise less than 2 °C (as set by the United Nations Framework Convention on Climate Change, UNFCCC) [31]. The results showed that technological changes alone cannot reach the 2 °C target. Only technological change with a 50% reduction in economic growth may come close to the goal.

The studies above focus on evaluating changes in household consumption patterns and include also economic growth or technological advances. However, what is lacking is a systematic evaluation of the emission mitigation potential of low-carbon behavior (in our case in China only) when compared to the situation where all countries emulate the lifestyle in developed countries. Different from previous models, the scenario model used in this paper is a semi-closed demand-driven model. This allows us to incorporate a natural dynamic link. Specifically, GDP in year *t* is determined by household consumption in year *t*. Consumption in year *t* in its turn is determined by GDP in the previous year *t*–1.

The paper connects the consumption-based emission approach with scenario analysis at the macro level. It focuses on the GHG emissions embodied in the global supply chains of final consumption. This means that we only take emissions into account that are generated in production processes. The direct emissions generated in the process of consuming the products (e.g., families driving their car or heating their home) are not included. The GHG emissions embodied in the global supply chains of final products account for 80% of the total emissions related to household activities [28]. Our analysis thus captures the majority of environmental impacts of final consumption.

The model used in this research deviates in one important aspect from econometric input-output models [32,33] and general equilibrium models [34,35]. That is, our model cannot capture the impact of price changes. Our model assumes that the relative prices across industries and countries remain constant. A consequence is that substitution between products due to price change is not included. On the one hand, if minimizing emissions is the objective in the process of substitution, an optimal solution with substitution cannot have more emissions than an optimal solution without substitution (rather less). In other words, our results are too pessimistic in the sense that emissions will be reduced more if substitution is allowed and if behavior is characterized by the objective of minimizing emissions. On the other hand, if Chinese households switch to low-carbon products and supply of these is limited, their price will increase. With nominal budget shares this implies smaller quantities of low-carbon products than in the no-substitution case. In other words, our results are too optimistic in the sense that reductions in emissions will less. These two cases illustrate that including substitution requires us to model the behavior of producers and consumers, which is beyond the scope of this paper.

When compared to dynamic CGE models, our input-output model is fairly simple and straightforward to apply. Instead of including economic behavior, we follow the “engineer’s view on economics” implying that the outcomes may need to be adapted when behavior (which is highly uncertain in the future and difficult to predict) is included. The advantage is that the model is straightforward to apply and that the results are tractable. CGE models typically require that numerous parameters (such as substitution elasticities) are given a numerical value. The values of most of the parameters are taken directly from the literature. CGE models are often more complex in structure which makes the results more difficult to interpret. The difficulty of predicting future economic behavior coupled with the possibility to interpret the emission results and to trace them to changes in consumption made that we have chosen to use an input-output model. With the dynamic link between consumption and GDP in the semi-closed input-output model, the environmental effects of different lifestyles are easily captured.

## 3 Data and model

There are a number of MRIO databases available including EXIOBASE [14], WIOD [36], Eora [37], the ICIO [38] and the GTAP database (with interpolation provided as part of work in the Global Carbon Project [39]). We use EXIOBASE because of its regional, sectoral and product detail, especially in Food and electricity generation categories, and its suitability for calculation of environmental impact of household behavior changes: EXIOBASE consists of a MRIO constructed for 44 countries (28 EU and 16 non-EU, including all major EU trading partners) and five Rest of the World regions (RoW), disaggregated into 200 products (including 17 products in Agriculture and 12 products in electricity generation). A full analysis of differences between MRIO databases can be found in Ref. [40]. In specific, we use data from EXIOBASE version 3.3 [14], since it covers the main GHG emission data (CO2, CH4, N2O, and SF6) at a high industrial resolution and thus is suitable for our footprint analysis. We take 2011, which is the most recent year in the database, as the base year.^6^ To model the consumption patterns and different lifestyles, the product-by-product world input-output tables from 1995 to 2011 are used for the estimation of the variables in the scenario model.

Table 1 presents a simplified world input-output table with n countries and every country has m products. Every row in the input-output table indicates the supply of outputs, which can be sold for intermediate use (in the blocks labeled Z) and for final use (in the blocks labeled F). Every column indicates the inputs for production, including intermediate inputs (in the blocks labeled Z) and value added inputs (in the blocks labeled v). The tables are expressed in monetary terms (in current, basic prices). The values are all in Euros.^7^

**Table 1:** A simplified world input-output table with n countries and m products per country.

	Intermediate use (m columns per country)			Final use (h columns per country)			Total
	1	…	n	1	…	n	
m products, country 1	$Z^{11}$	⋯	$Z^{1n}$	$F^{11}$	⋯	$F^{1n}$	$x^{1}$
…	⋮	⋱	⋮	⋮	⋱	⋮	⋮
m products, country n	$Z^{n1}$	⋯	$Z^{nn}$	$F^{n1}$	⋯	$F^{nn}$	$x^{n}$
Value added	${\left(v^{1}\right)}^{\prime }$	⋯	${\left(v^{n}\right)}^{\prime }$				
Output	${\left(x^{1}\right)}^{\prime }$	⋯	${\left(x^{n}\right)}^{\prime }$				
${\text{CO}}_{2}$ emissions	${\left(e^{1}\right)}^{\prime }$	${\left(e^{.}\right)}^{\prime }$	${\left(e^{n}\right)}^{\prime }$				

The superscripts and subscripts denote country and product, respectively. For example, $Z^{rs}$ is an m×m matrix and its element $z_{ij}^{rs}$ indicates the inputs of product i in country r for the production of product j in country s.^8^ Note i,j=1,2,…,m. $F^{rs}$ is an m×h matrix and its element $f_{ik}^{rs}$ indicates the final demand of product i from country r required by type k in country s, where k indicates the final demand type, covering household consumption, consumption of non-profit organizations, government consumption, gross fixed capital formation merged with changes in inventories (k=1,2,3,4).^9^ Finally, $v^{r}$ is an m-element vector and its typical element $v_{i}^{r}$ gives the value added in product i in country r. $e^{r}$ is an m-element vector and its typical element $e_{i}^{r}$ gives the emissions generated in the production of product i in country r.

Using the information contained in global input-output tables structured according to Table 1, the global input-output model can be formulated as

(1) $$\left[\begin{array}{ccc} Z^{11} & \cdots & Z^{1n}\\ \vdots & \ddots & \vdots \\ Z^{n1} & \cdots & Z^{nn} \end{array}\right]1+\left[\begin{array}{ccc} F^{11} & \cdots & F^{1n}\\ \vdots & \ddots & \vdots \\ F^{n1} & \cdots & F^{nn} \end{array}\right]1=\left[\begin{array}{c} x^{1}\\ \vdots \\ x^{n} \end{array}\right]$$

where 1 is a vector of ones of appropriate size used for summation. Define the input coefficient as

$$A=\left[\begin{array}{ccc} A^{11} & \cdots & A^{1n}\\ \vdots & \ddots & \vdots \\ A^{n1} & \cdots & A^{nn} \end{array}\right]$$

where $A^{rs}$ is the direct input coefficient matrix from country r to country s, obtained as $A^{rs}=Z^{rs}{\left({\hat{x}}_{s}\right)}^{-1}$. Then Equation (1) can be solved as

$$\left[\begin{array}{c} x^{1}\\ \vdots \\ x^{n} \end{array}\right]={\left(\left[\begin{array}{ccc} I & \cdots & 0\\ \vdots & \ddots & \vdots \\ 0 & \cdots & I \end{array}\right]-\left[\begin{array}{ccc} A^{11} & \cdots & A^{1n}\\ \vdots & \ddots & \vdots \\ A^{n1} & \cdots & A^{nn} \end{array}\right]\right)}^{-1}\left[\begin{array}{ccc} F^{11} & \cdots & F^{1n}\\ \vdots & \ddots & \vdots \\ F^{n1} & \cdots & F^{nn} \end{array}\right]1$$

In aggregated matrix format, we have $x={\left(I-A\right)}^{-1}F1=LF1$, where $L={\left(I-A\right)}^{-1}$ is the Leontief inverse. Denote $\rho^{r}$ as the emission intensity per unit of output in region r, with $\rho_{j}^{r}=e_{j}^{r}/x_{j}^{r}$. Then the global ${\text{CO}}_{2}$ emissions generated along production chains can be calculated as: $e={\left(\hat{\rho }\right)\left(I-A\right)}^{-1}F1=\left(\hat{\rho }\right)LF1$**.** Similarly, denote the $\vartheta_{j}^{r}=v_{j}^{r}/x_{j}^{r}$ as the value added coefficient, then the value added vector can be obtained as $v={\left(\hat{\vartheta }\right)\left(I-A\right)}^{-1}F\tau =\hat{\vartheta }LF1$.

In our scenario analysis, changes in lifestyles will induce changes in the final demand matrix F. Other elements of the scenario will affect the structure of production, leading to changes in the direct input coefficient matrix A. Changes in the emission intensity of production will be reflected by changes in the emission coefficient vector ρ. By incorporating the time dimension, we have the value added vector and emissions in year t calculated as

$$v_{t}={\left({\hat{\vartheta }}_{t}\right)\left(I-A_{t}\right)}^{-1}F_{t}1={\hat{\vartheta }}_{t}L_{t}F_{t}1$$

$$e_{t}={\left({\hat{\rho }}_{t}\right)\left(I-A_{t}\right)}^{-1}F_{t}1=\left({\hat{\rho }}_{t}\right)L_{t}F_{t}1$$

### 3.1 Analytical framework

**Fig. 1:**
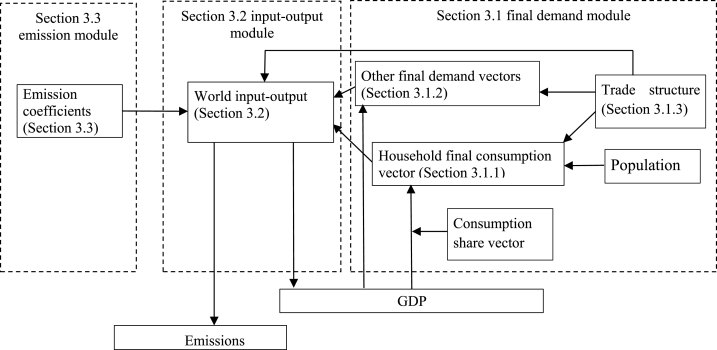
Analytical framework.

Fig. 1 summarizes the analytical framework in this paper. The details (including mathematical formulations) are given in Appendix B and some of the blocks refer to specific sections in that appendix. Four key elements are modeled in the environmentally extended multiregional input-output model and discussed below. These are: household final consumption for each product (m=200) in each country (n=49); similar vectors for the other three types of final demand vectors; the world input-output structure; and product- and country-wise emission coefficients. The model is estimated on the basis of annual data for the period 1995–2011, after which projections for the emissions embodied in final consumption are made for the years up to 2030.

1. For all countries and all products, household consumption is calculated in three steps and on a per capita basis (using population data from the United Nations Population Division). First, the total consumption (per capita) is modeled as a fraction of previous year’s GDP (per capita). Second, we use the “world Engel curves” to determine the share of total consumption expenditures that is spent on a particular product *i* (=1, …, 200). The estimation of the world Engel curves is described in Appendix A. The idea behind using the world Engel curves is that the consumption shares (together characterizing the product mix of consumption) are dependent on total consumption expenditures per capita. The Engel curve for a product gives the consumption share of that product for every level of total consumption. Our assumption is that these curves apply globally, hence the term *world* Engel curves. This implies that if poor countries increase their average income, the consumption shares will change accordingly. Consumers will emulate the lifestyle of richer countries when they get richer themselves [3]. Third, these consumption patterns in each country are further split to indicate the country from which the products are sourced. For example, in the second step we have determined the consumption expenditures per capita in Germany on processed rice, the third step splits this into Italian rice, Indian rice, et cetera.
2. Other final demands are not directly influenced by living standards, which is why they are estimated in a different way. Specifically, the expenditures for any product *i* by non-profit organizations, for government final consumption, and for gross fixed capital formation and inventory changes are modeled proportional to previous year’s GDP level. The composition of product shares in each of these three final demand vectors is fixed to the shares of the base year (2011).
3. For the world input-output structure, the composition of the intermediate inputs that are necessary per unit of production (for any product in any country) is assumed to be fixed over time. This means that the matrix with direct input coefficients is assumed constant ($A_{t}=A$). As a robustness check, however, we will include changes in the relative importance of countries as the origin of China’s inputs and products for final use in one of the scenarios. For both cases, the assumptions imply that the value added coefficients are fixed over time ($\vartheta_{t}=\vartheta$).
4. The emission coefficients are assumed to be constant for the projection period, considering that the relative emission changes caused by different lifestyles is the main focus of the study while detailed modeling of emission intensity changes is not. As a robustness check, we also run a scenario where the historical trends in the emission coefficient changes of the top-5 emission-intensive products are extrapolated. The emission coefficients of other products are kept fixed. These top-5 products are: Cement, lime and plaster; Electricity by coal; Electricity by gas; Electricity by petroleum and other oil derivatives; and Steam and hot water supply services. They contributed 35% of global emissions in 2011.

Based on year t’s world input-output table, the scenario assumptions, and the exogenous variables, emissions and GDP in year t are calculated. This then determines household final consumption in year t+1, after which the emissions and GDP in year t+1 are calculated. This again yields consumption in t+2, and so forth. Applying this procedure 19 times (starting in the base year 2011), we arrive at the projection for 2030.^10^

### 3.2 Scenario description

Essentially, we consider two different scenarios. In the baseline scenario, all countries follow the world Engel curve when determining the mix of consumption goods. This means that countries emulate the lifestyle of richer countries when they become richer themselves. This baseline scenario is indicated by BL. The second scenario (indicated by LC) supposes that Chinese households adopt low-carbon behavior. To check the robustness of our results, we also apply BL and LC to the case where China decreases the import shares. This case with trade pattern changes is indicated as TP. Table 2 summarizes the four possible scenarios). BL and BLTP represent two baseline scenarios where people tend to follow the lifestyles currently adopted by households in richer countries, while LC and LCTP indicate two low-carbon scenarios in which Chinese household adopt low-carbon behaviors.

**Table 2:** Main characteristics of scenarios to 2030 of global carbon footprint of final demands.

		Assumptions		
		Household final consumption pattern	Trade structure in final demands	Trade structure in intermediate inputs
Baseline Scenario (Emulation)	BL	Consumption composition changes with the growth of total expenditure along the “world Engel curves”	Same as in the base year	Same as in the base year
Low-carbon scenario	LC	Compared to scenario BL, the level of household consumption in China will reduce. See Table 3 for details.	As in BL	As in BL
Baseline scenario with trade pattern changes	BLTP	As in BL	Import shares in China decease by 1% per year	Import shares in China decrease by 3% per year
Low-carbon scenario with trade pattern changes	LCTP	As in LC	As in BLTP	As in BLTP

All scenarios share common assumptions for other final demands, for the world input-output model, and for the emission coefficients. In the low-carbon scenarios (LC and LCTP), a dematerialized lifestyle behavior is preferred by Chinese household. Households are assumed to save and reuse goods and materials, and the saved expenditures are re-spent on goods from other low-carbon categories. In the TP scenarios (BLTP and LCTP), trade structure changes are assumed for China. Imported goods are replaced by domestically produced goods at a rate of 1% (for final goods) or 3% (for intermediate goods) per year. The low-carbon and trade pattern change scenarios are described in more detail in this section.

#### 3.2.1 Low-carbon scenario

In the low-carbon scenario, the assumptions are the same as in the baseline scenario, except for the Chinese household consumption pattern which is modeled to follow a low-carbon behavior. For this, we first allocate the 200 products in EXIOBASE in one of seven categories, as suggested by Ref. [29]. These are: Clothing, Construction, Food, Manufactured products, Mobility, Services, and Shelter (see Appendix C for the concordance table between the 200 products and the seven categories).^11^ Next, the potential changes that are feasible in the low-carbon scenario (when compared to the baseline scenario) are obtained for each category from case studies and experts’ estimations in the literature.

Broadly speaking, there are two ways through which changing lifestyles could contribute to the low-carbon goal. The first is via reducing the consumption level. This can be achieved by either reducing avoidable waste (such as reducing food waste) or increasing the lifetime of final products [42]. The second is via a shift of consumption goods to products with a lower global carbon footprint. For example, substituting beef for low-carbon meat, such as pork or chicken. In this paper, we focus on the first way of changing towards a low-carbon lifestyle. This is relatively simple to implement for consumers and avoids the modeling with consumers’ preferences. For the construction of low-carbon behavior, the case studies of the Waste and Resources Action Programme (WRAP) serve as a starting point. The potential consumption reductions are taken from the case studies and other literature, and are given in Table 3. Note that no reductions are listed for Services, which is because no assumptions were found in the literature for this category. Because the emission multipliers for services are very low, this approach will not affect the results.

**Table 3:** Low-carbon behavior setting.

Category^a^	Description	Product implication (change in $f_{1\left(t\right)}^{c}$)	Sources
Clothing	Expanding lifetime; reduce the rate of purchases and disposals	Reduce clothing-related products by 30%	WRAP research shows that around 30% of clothing in the wardrobes has not been worn for at least a year; nearly 30% of non-clothing textiles were estimated to be reusable
Construction	Reduce waste	20% of material cost reduction: clay products, cement, steel, stone, sand and clay, wood and non-metallic mineral products	The WRAP case study showed that in the case of household construction, well-prepared design in advance can reduce waste and achieve 20% saving of material cost on site (Langdon, 2009, p58) [43]
Food	Eliminate avoidable waste	Avoidable food waste percentages for industrialized Asia are: Cereals 20%; Roots and tubers 20%; Oilseeds and Pulses 4%; Fruits and vegetables 15%; Meat 8%; Fish and seafood 8%; and Milk 5%	Avoidable food waste corresponding to each food products are obtained from [16]
Manufactured products	Less plastic and chemical products + minimizing purchases of personal and household electronic products	23% reduction in plastic and other chemicals products; 23% reduction in machinery and electric apparatuses	Research carried out by WRAP showed that 23% of the Waste Electrical and Electronic Equipment (WEEE) could be re-used
Mobility	Less mobility (collective transport)	50% reduction of all products related to mobility	Based on the scenario setting in Ref. [44]: reducing needs for mobility can be achieved by working from home, living close to work, sustainable mobility education; car sharing (such as Didi and Uber) is increasing in China
Shelter	Expanding the lifetime of furniture	30% reduction in furniture	The Furniture Re-use Network (FRN) showed that around 30% of the bulky waste collected from households can be reused
	Energy-conscious behavior	Decrease electricity use by 14%	Random control trials conducted by Ref. [45] showed that energy-saving education could save 14% of household electricity use

a The consumption of services products is not reduced.

The saved expenditures through reducing and changing consumption are re-spent by households on other products in the same category (on which savings are not possible). This re-spending is done in proportion to the existing composition (of the products that are involved). However, since household consumption of all the products classified in category of Clothing, Construction and Mobility can be saved, the expenditure savings cannot be re-spent in the same category. In this case, the expenditure savings are assumed to be re-spent in the “clean” category, that is, Services. Further details (and a mathematical exposition) are given in Appendix B.4.

It should be stressed that shifting final demand to products with a lower global carbon footprint (such as substituting beef by pork) is not considered in the model. Therefore, this research evaluates the emission reduction by Chinese household through avoiding wastes and increasing the lifetime of products. This is a clear lower bound for the actual reduction that can be achieved.

#### 3.2.2 Trade structure change scenario

Considering China’s recent policy of stimulating domestic consumption and its trade pattern change over last decades, it is likely that imported share in final demand and intermediate inputs will continue decreasing, implying an increasing share of domestically produced goods. As for the final demand, the share of domestic products will increase, due to China’s transition from growth mainly relying on investment and exports to growth based on domestic consumption and investment. As for the intermediate inputs, the efforts of upgrading China’s position in global value chain and decreasing the share of processing trade in total output all contribute to the decreasing share of imported inputs. Therefore, we also analyze the BL and LC scenarios with trade pattern changes, namely BLTP and LCTP scenarios. In both scenarios, we assume that the imported share of final products and intermediate inputs for China will continue along the historical downward trend and decrease by 1% and 3% per year and reach 3.7% and 6% in 2030, respectively.

For other countries, the trade structure of final demand and intermediate inputs are assumed to remain constant as in year 2011. This implies that we assume product competitiveness of China will keep the same as in year 2011 and other countries will not substitute Chinese produced products with those produced by other countries. For the detailed modeling description of trade structure change scenario, please refer to Appendix B.

## 4 Results

### 4.1 The effects of emulation of lifestyles and the low-carbon lifestyle in China

#### 4.1.1 The effects of emulation of lifestyles on Chinese household consumption

Before evaluating the environmental performance, we analyze the effects of the different lifestyles on Chinese household consumption. First, over time, lifestyles emulate globally and thus also in China, affecting Chines household consumption. Emulation means that, because GDP grows, the total amount of consumption expenditures grows and the Chinese consumption pattern will move along the world Engel curves, as estimated for each of the 133 products for which household demand is positive. Chinese households are expected to adapt their consumption pattern and become more similar to richer countries. In the baseline scenario (BL), the total Chinese household consumption grows from 1498 billion euros in 2011–4255 billion euros in 2030.

**Fig. 2:**
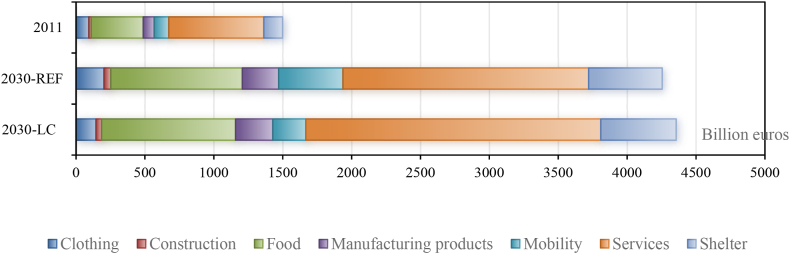
Final demand structure of Chinese households.

Fig. 2 summarizes the composition of Chinese household consumption over the seven major product categories. Comparing the columns for 2011 and for the baseline scenario (BL) in 2030 shows the effects of emulation (Table 4). We find that the share of consumption expenditures spent on Food drops by 3% points (from 25% to 22%) and on Clothing by 1% point. The shares for Mobility and Shelter in total household expenditures will each increase by 4% points from 2011 to BL in 2030. These results are consistent with the literature [3,6]. In particular, an increase in household consumption induces increased demand for heating and space cooling (in Shelter) and for cars and long-distance travelling (in Mobility). The share spent on Services drops by 4% points (from 46% to 42%). As indicated in Appendix A, Chinese households spent relatively much on Services in 2011, which will reduce after emulation of their lifestyle according to the world Engel curves.^12^

**Table 4:** Final demand structure change of Chinese households (Unit: Billion euros).

	Low-carbon effect 2030			
	LC minus BL		LCTP minus BLTP	
		%		%
Clothing	−58	−1	−80	−1
Construction	−9	0	−13	0
Food	18	0	20	0
Manufacturing products	6	0	8	0
Mobility	−227	−5	−328	−6
Services	358	7	501	7
Shelter	13	0	15	0
Total	101	0	123	0

Note: Columns with % represent the share of each category in the total consumption expenditure.

The per capita household consumption increases from 1090 euros in 2011–2935 euros in 2030. This means that the per capita level of consumption China in 2030 would be similar to that of Bulgaria in 2011 (with 2898 euros). It turns out that the consumption pattern of Chinese households is indeed similar to that of Bulgarian households.^13^

It should be mentioned that Table 4 only gives the shares at category level, whilst many changes take place within a category (i.e. at the product level). Appendix D presents the full details. For example, within the category Food, the share (in the expenditures on Food) of ‘Vegetables, fruits and nuts’ drops by 13% points from 23% to 10%. The total share of “milk and meat” (i.e. ‘Raw milk’, ‘Dairy products’, ‘Products of meat’) rises by 23% points from 5% to 28%. This indicates that small changes at category level may hide relatively large but opposite changes at product level.

#### 4.1.2 The effects of a low-carbon lifestyle on Chinese household consumption

Secondly, we compare the pattern of household consumption in the baseline scenario (BL) with the low-carbon scenario (LC). In Table 4, the column LC and the differences of expenditures and shares between LC scenario and BL scenario are given in the column “LC minus BL”. We find that the share of expenditures spent in 2030 on Services is 7% points higher (42% in BL and 49% in LC). At the same time, the share for Mobility is substantially (5% points) smaller and the share for Clothing a bit (1% point). As a robustness check, we also examined the effect of introducing the LC lifestyle in the case in which the trade patterns also change (the TP scenario). The difference between the LC variant of the TP scenario (indicated as LCTP) and the baseline TP scenario (BLTP) are given in the column “LCTP minus BLTP”. The results are very similar to those in the columns “LC minus BL”.

If the low-carbon lifestyle will be adopted by Chinese households, the total consumption expenditures in 2030 will be roughly 2% higher in comparison to the BL scenario (4255 and 4356 billion euros in BL and LC, respectively). This is caused by the fact that the reduced expenditures for one product (because of the low-carbon lifestyle) are spent on another product. For most cases this will be a product in the same category. The reduced expenditures in Mobility, Clothing, and Construction, will be spent on products in the category Services. The domestic value-added per euro of Services production, however, is generally higher than for production in other categories. Consequently, the low-carbon scenario leads to a higher GDP in China and thus to more household consumption.

#### 4.1.3 The effects on GHG footprints of Chinese households

**Fig. 3:**
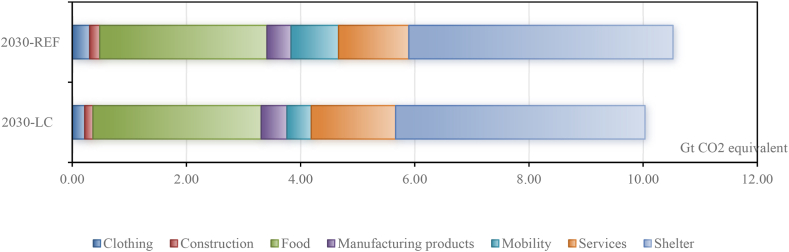
Global carbon footprint of Chinese households in 2030.

The GHG footprints of the seven consumption categories are given in Fig. 3 for the BL and the LC scenario, and in Table 5 for the differences between scenarios. The footprints give the GHG emissions anywhere in the world that are embodied in the consumption by Chinese households. The footprints can be determined at the product level, at the category level, or at the level of total household consumption. The results for the BL scenario in Fig. 3 show that, due to total expenditure growth and emulation of lifestyles, the GHG footprint of total household consumption grows from 1.72 Gt CO_2_-equivalent in 2011 to 10.52 Gt in 2030. Adopting a low-carbon lifestyle by Chinese households reduces the 2030 footprint by 5% (from 10.52 Gt in BL to 10.03 Gt in LC, or from 14.89 Gt in BLTP to 14.10 Gt in LCTP).

**Table 5:** Global carbon footprint change of Chinese households in 2030 (Unit: Gt CO_2_-equivalent).

	Low-carbon effect			
	LC minus BL		LCTP minus BLTP	
		%		%
Clothing	−0.09	−1	−0.13	−1
Construction	−0.03	0	−0.05	0
Food	0.02	2	−0.01	1
Manufacturing products	0.03	0	0.03	0
Mobility	−0.41	−4	−0.62	−4
Services	0.25	3	0.37	3
Shelter	−0.26	0	−0.39	0
Total	−0.49	0	−0.79	0

Note: Columns with % represent the shares of footprint by each category in the total consumption footprint.

The differences for 2030 in the consumption expenditures in Mobility lead to the largest reduction in emissions, those in Services to the largest increase in emissions. Although the absolute reductions in Mobility (−0.41 Gt) and Clothing (−0.09 Gt) are not extremely large, it should be stressed that they are substantial in relative terms. The 2030 footprints for Mobility and for Clothing are almost 50% and almost 30% smaller, respectively.

The results for Mobility, Clothing, and Services correspond to the changes in consumption expenditures as reported in Table 4. That is, lower (or higher) consumption leads to lower (higher) emissions. This is not the case for Shelter, where the expenditures are 13 billion euros higher in LC than in BL, but the footprint is 0.26 Gt smaller. This is because in the low-carbon lifestyle scenario (Table 3), households shift away from consumption of products related to furniture and electricity use. The saved money is re-spent on other products in the Shelter category. The products that are used less (such as electricity by coal, see Appendix D) are generally more emission-intensive than the products that are used more.

Table 5 also shows the difference in the overall emission multiplier of Chinese household consumption. It measures the kilograms of global emissions per euro of Chinese household consumption (either at product, category, or overall level).^14^ The overall multipliers are 2.47 kg/euro (=10.52 Gt divided by 4255 billion euros) in BL and 2.30 kg/euro in LC. The low-carbon lifestyle makes the consumption structure less emission intensive, not only across categories, but also within categories. Across categories, the relative share of Services in the total consumption of 2030 is 7% higher in LC than in BL (Table 4), but the emission multiplier for the category Services is much smaller than for other categories. Within categories, within Shelter we observed that products with a lower share (in LC compared to BL) have a higher emission multiplier than products with a higher share. The consequence is that the emission multiplier for the category Shelter is lower (8.66 and 7.98 kg/euro in BL and LC, respectively).

### 4.2 The global economy and its emissions

The results so far were obtained for a model in which all countries have GDP growth and emulation of their lifestyle according to the world Engel curves. For China, we also included a change to a low-carbon lifestyle. The previous section focused on the effects on expenditures of Chinese households and the consequences for global emissions. In this section, we consider all effects at a global scale. That is, whereas Table 5 gives the global emissions embodied in Chinese household consumption, Table 6 presents the emissions embodied in Chinese household consumption, other Chinese final demands and foreign final demands combined.

**Table 6:** Projected global GDP and GHG emissions under different scenarios.

				Low-carbon effect	
		BL	LC	LC minus BL	LCTP minus BLTP
GDP (Billion Euros)	2011	52,304			
	2030	82,429	82,672	0.3%	0.3%
GHG emissions (Gt CO_2_-equivalent)	2011	40.1			
	2030	76.3	76.1	−0.2%	−0.5%

Table 6 shows that adopting a low-carbon lifestyle in China stimulates global GDP a little. Under the baseline scenario (in which all countries emulate their lifestyles), global GDP grows steadily from 52 trillion euros in 2011 to 82 trillion euros in 2030. Under the low-carbon scenario in China, global GDP is 0.3% (243 billion euros) larger in 2030 than under BL.^15^ Recall from Table 4 that China’s household expenditures in 2030 are “only” 101 billion euros larger under LC than under BL. Changing the lifestyle in China affects foreign production through extra imports of final products and intermediate inputs for Chinese production.

The low-carbon lifestyle in China not only benefits global GDP a little but also global GHG emission mitigation. Under BL, global emissions will increase from 40.1 Gt in 2011 to 76.3 Gt in 2030. Under the LC scenario, the emissions will increase 0.2 Gt less. Similar results are found for the effect of adopting a low-carbon lifestyle when trade pattern changes are included in the scenarios (i.e. comparing LCTP and BLTP).

The reduction in global emissions that is achieved by Chinese households adopting a low-carbon lifestyle is very small (0.2 Gt, which is only 0.3% of the global emissions in 2030). As an indication, meeting the goals of the Paris Agreement requires the global emissions to decline from approximately 36 Gt in 2016 to 21 Gt in 2030, to 11 Gt in 2040 and to 5 Gt in 2050 [46]. We have also run a scenario in which all countries adopt the same low-carbon lifestyle, using the assumptions as sketched in Table 3. This leads to 71.7 Gt of global GHG emissions in 2030, which is 4.6 Gt less than the 76.3 Gt in the baseline scenario. The message is very clear, the adoption of the mild form of lifestyle change that we have included in our low-carbon scenario leaves the world far away from the emission target (of 21 Gt in 2030) necessary for the 2 °C goal.

Observe that Table 5 reports that the global emissions embodied in the Chinese household consumption in 2030 are 0.5 Gt lower if these households adopt a low-carbon lifestyle. This is more than the 0.2 Gt difference in global emissions reported in Table 6. This reveals that the induced effect offsets a large part of the emission mitigation effect of low-carbon behavior adoption by Chinese households. Because GDP in China in 2030 is higher in LC than in BL, other Chinese final demands (such as investments and government expenditures) are also higher. It turns out that the footprint of these other final demand increases by 0.4 Gt. Another (much smaller) induced effect runs through foreign final demands, which causes somewhat lower global emissions under LC.

### 4.3 Emission changes at the product level

At the product level, the low-carbon lifestyle scenario for Chinese households will help reduce emissions in most products. Table 7 shows that the largest emission differences between LC and BL are witnessed for products in the categories Shelter and Mobility. This is related to the large saving rates in Mobility (see Table 3) and large emission multipliers of products in Shelter. The three products with the largest emission reduction are part of Shelter and for these three products together emissions in 2030 are 366 Mt CO_2_-equivalent lower in LC than in BL. Re-spending of savings increases emissions in other products. The three products that exhibit the largest increases in emissions in the LC scenario, lead together to 174 Mt CO_2_-equivalent more than in BL.

**Table 7:** Emission differences at the product level due to adoption of the low-carbon lifestyle in China, 2030 (Unit: Gt CO_2_-equivalent).

No	Product	Category		Emission difference	
148	Steam and hot water supply services	Shelter		−0.258	
28	Crude petroleum and services related to crude oil extraction, excluding surveying	Shelter		−0.056	
128	Electricity by coal	Shelter		−0.052	
157	Railway transportation services	Mobility		−0.025	
14	Raw milk	Food		−0.022	
162	Air transport services	Mobility		−0.021	
147	Distribution services of gaseous fuels through mains	Shelter		−0.019	
158	Other land transportation services	Mobility		−0.014	
161	Inland water transportation services	Mobility		−0.013	
67	Motor Gasoline	Mobility		−0.009	
⁝	⁝	⁝	⁝		
62	Paper and paper products	Shelter		0.010	
49	Processed rice	Food		0.010	
150	Construction work	Construction		0.013	
164	Post and telecommunication services	Services		0.014	
8	Crops nec	Food		0.015	
22	Other Bituminous Coal	Shelter		0.019	
104	Basic iron and steel and of ferro-alloys and first products thereof	Construction		0.021	
190	Food waste for treatment: landfill	Shelter		0.047	
101	Cement, lime and plaster	Construction		0.054	
1	Paddy rice	Food		0.073	

Note: This table lists the ten products with the largest reductions in emissions and the ten products with the largest increases in emissions, when comparing the LC with BL scenario.

Although the mitigation potential of low-carbon behavior is not very large, small changes in lifestyle can still lead to sizeable emission reduction if the products can be well selected. It may be helpful for consumers to know the emission impacts at product level. Table 8 gives the 15 products with the largest emission multipliers for their consumption and a household consumption share larger than 0.5% (=1/200, the average consumption share) in 2011. The top 6 products all belong to Shelter, where coal-fired electricity stands out. One euro of its consumption leads to 27 kg of CO_2_-equivalent emissions. Also the emission multipliers for the distribution of electricity, gaseous fuels, and water (ranking at positons 2–4 in Table 8) are very large. This indicates that substantial reductions in emissions can be achieved if consumers manage to reduce electricity, water and energy use. Moreover, since “Electricity by Coal” is widely used as input in production, cleaning the generation of coal-fired generation of electricity will decrease almost every emission multiplier.

**Table 8:** The top 15 products with the largest emission multipliers for their consumption by Chinese households.

No.	Product	Category	Multiplier	Consumption share
128	Electricity by coal	Shelter	26.91	0.74%
141	Distribution and trade services of electricity	Shelter	15.42	0.77%
147	Distribution services of gaseous fuels through mains	Shelter	6.64	0.55%
149	Collected and purified water, distribution services of water	Shelter	5.05	0.64%
96	Rubber and plastic products	Shelter	2.87	0.60%
90	Chemicals nec	Shelter	2.81	1.50%
150	Construction work	Construction	2.78	1.13%
67	Motor Gasoline	Mobility	2.53	1.01%
3	Cereal grains nec	Food	2.53	0.72%
53	Fish products	Food	2.29	6.43%
157	Railway transportation services	Mobility	2.04	0.55%
175	Health and social work services	Services	1.78	6.07%
125	Furniture; other manufactured goods n.e.c.	Shelter	1.69	2.53%
120	Electrical machinery and apparatus n.e.c.	Manufactured products	1.68	2.46%
57	Leather and leather products	Clothing	1.65	1.15%

Note: multipliers are in kg of CO_2_-equivalent per euro of consumption of the corresponding product (based on 2011 data).

## 5 Conclusion and policy implications

In this paper we have used the input-output model to analyze the effects of the adoption by Chinese households of a low-carbon lifestyle. Our scenario model estimates final demands and corresponding GHG emissions for 49 countries year by year from 2012 to 2030.

Two features make our model different from the standard model. One is that we have assumed that all countries copy the consumption pattern of richer countries once they become richer themselves. This emulation of lifestyles is modeled by assuming that all countries follow world Engel curves, implying that the composition of household consumption changes with changing total expenditure on consumption. These world Engel curves were estimated for 200 products using annual EXIOBASE data for 49 countries from 1995 to 2011. The second feature is that the model assumes that this year’s consumption depends on last year’s GDP, which makes it dynamic.

The baseline scenario (BL) assumes that all countries in the world adopt the emulation of lifestyles. The low-carbon scenario (LC) assumes that Chinese households adopt a low-carbon lifestyle by saving and reusing goods and materials. This is achieved by reducing avoidable waste (such as reducing food waste) or increasing the lifetime of final products [42]. For the specific assumptions under low-carbon behavior, the case studies of the Waste and Resources Action Programme (WRAP) served as a starting point.

The results in this paper led to the following two key conclusions. First, the emulation of consumption patterns increases GHG emissions more than it increases GDP. It follows from the BL results in Table 6 that GDP increases on average by 2.4% per year whilst the emissions grow annually with 3.4%. On the one hand, economic growth brings along emission growth because of more economic activity. On the other hand, economic growth comes with extra emission growth because countries mimic richer countries and their consumption patterns, which are more emission-intensive.

Second, adopting a mild low-carbon lifestyle by households helps only little in terms of reducing GHG emissions. If only Chinese households adopt the low-carbon lifestyle, the global emissions in 2030 embodied in Chinese household consumption will be 0.5 Gt CO_2_-equivalent lower than in the baseline, according to Table 5. Because the low-carbon lifestyle increases China’s GDP, also other Chinese final demands increase implying an induced effect that increases emissions. The global net effect is a reduction of 0.2 Gt CO_2_-equivalent (Table 6), which is a reduction of 0.3% when compared to the emissions in 2030 for the baseline scenario. If all countries adopt the same low-carbon lifestyle, the reduction in 2030 is 4.6 Gt CO_2_-equivalent (which is 6.2% of the emissions in 2030 under BL).

The reduction in GHG emissions achieved from households adopting a low-carbon lifestyle is very low. The key message is that avoiding waste helps to reduce emissions, but only marginally. Looking at household behavior, it should be stressed that the scope of our LC scenario is rather limited. It did not capture any substitution of products with a large emission footprint for products with a lower footprint (e.g., substituting beef for chicken, or a vegetarian diet). Another aspect is that the scenario only worked with options that are available now and not with expected options (e.g., fully decarbonized heating of houses in the future). The same holds for changes in technology (although major breakthroughs that can be implemented at a large scale are not foreseen before 2030). Eliminating avoidable waste is just a simple and small change in lifestyle and the results may thus be expected to give a lower bound for emission savings that might be achieved from a more radical change in lifestyle by Chinese households.

A second reason why the differences in GHG emissions between the scenarios were very small, is that our scenario includes a rebound effect. We have assumed that the money that households save from avoiding waste and increasing the lifetime of products is re-spent on other products. In particular, savings in the categories Clothing, Construction and Mobility are re-spent in the category Services. Savings in the other categories (Food, Manufactured products, Shelter) are re-spent by households on other products in the same category. The size of the rebound effect is considerable. The savings led to a total reduction of emissions in 2030 by 0.6 Gt CO_2_-equivalent (0.5 Gt by the products in the top 10 in Table 7) and the re-spendings led to 0.4 Gt CO_2_-equivalent (0.3 Gt by the products in the bottom 10) higher emissions in that year. Although we do not specify an alternative option for the re-spending of the savings, it is clear that this may well lower the rebound effect. For example, savings might be used to work a bit less. As a consequence, the gains from adopting a low-carbon lifestyle will then increase.

A third reason was the induced effect, which in the case of China offset the reduction in emissions. Because the adoption of a low-carbon lifestyle increased Chinese GDP, next year’s final demands increased. This held not only for total consumption but also for other final demands (such as investments and government expenditures). The increases in other final demands led to a growth in emissions. An alternative to avoid the induced effect is to spend the GDP growth on subsidizing mitigation efforts, for example.

The message from our results is crystal-clear that to make “easy” behavior changes helpful to contributing to emission mitigation, efforts should made to get rid of the rebound and induced effects. On the one hand, household in all countries should oppose consumerism, insist to sustainable consumption and promote sustainable development. Lenzen et al. (2021) suggest that in particular the richer countries “scale down less necessary forms of production and consumption, reducing aggregate energy and resource use to enable rapid mitigation” [47]. On the other hand, part of the GDP growth should be dedicated to subsidizing mitigation efforts and developing low-carbon technologies, instead of focusing on expanding reproduction.

## Data availability statement

The model results are calculated based on the following data sources: EXIOBASE (https://www.exiobase.eu), China Statistical Yearbook (http://www.stats.gov.cn/sj/ndsj/), World Population Prospects - Population Division, United Nations (https://population.un.org/wpp/), WRAP (http://www.wrap.org.uk/).

## CRediT authorship contribution statement

**Bingqian Yan:** Writing – review & editing, Writing – original draft, Formal analysis, Conceptualization. **Erik Dietzenbacher:** Writing – review & editing, Supervision. **Bart Los:** Writing – review & editing, Supervision.

## Declaration of competing interest

The authors declare that they have no known competing financial interests or personal relationships that could have appeared to influence the work reported in this paper.

## Footnotes

1 The GHG emissions generated in China accounted for 23% of global emissions in 2012 according to EDGARv4.2FT2012 [10].

2 See https://data.worldbank.org/indicator/NY.GDP.PCAP.CD?end=2020&start=1960&view=chart.

3 https://cciced.eco/climate-governance/how-is-progress-in-chinas-1n-policy-framework/.

4 The literature that studies the relationship between environmental quality and income growth primarily focuses on examining the Environmental Kuznets Curve hypothesis and finds mixed empirical results [[19], [20], [21]].

5 This is also advocated by NDRC (2022) [9], which states that “[b]y 2025, the concept of green consumption will be deeply rooted in people’s mind, extravagance and waste will be effectively curbed, the market share of green and low-carbon products will increase significantly, major achievements will be made in the green transformation of consumption in key areas, green consumption patterns will be widely implemented, and a green, low-carbon and circular consumption system initially formed.”

6 In recent public versions of EXIOBASE, MRIO tables were updated to 2016 in version 3.7 (2019 release) and 2022 in version 3.8 (2020 release), respectively. The energy/emission data were only updated to 2015 at detailed level. However, these new versions rely heavily on “now-casting” economic structure (and environmental extensions) based on trade and macro-economic data, with no incorporation of more recent official SUT or IOT data than for 2011. As such, the uncertainty relative to official data at transaction level would be considerably higher. Since our research is about to simulate households’ behavior change and analyze its environmental impacts, it has a high demand on the accuracy of the base year data, especially the transactional flows. Therefore, we finally chose version 3.3 for analysis.

7 The main data source for the database construction is the United Statistics Division (UNSD) – National Accounts Statistics: Main Aggregates and Detailed Tables (United Nations Statistics Division - National Accounts 2013). These tables provide a complete and consistent set of time series from 1970 onwards of main national accounts aggregates for more than 200 countries/regions (http://unstats.un.org/unsd/snaama/). The UN tables are available in current USD and are converted to Euros using the annual period-average market exchange rate of each year [41].

8 Matrices are indicated by bold capital letters; vectors are columns by definition and are indicated by bold lowercase letters; scalars by italicized lowercase letters. A prime indicates transposition and a hat (or a circumflex) indicates a diagonal matrix with the elements of a vector on its main diagonal and all other entries equal to zero.

9 Changes in inventories are relatively small and random.

10 The reason for only choosing these five industries is twofold. First, they are among the top-10 emission-intensive products for most countries. The large emission coefficients for these products implies considerable opportunities to reduce emissions. A 5% reduction in the emission coefficient for these products yields a much larger effect than a 5% reduction in the coefficient for other products. Second, these products are used where they are produced and are less likely to be traded. Changes in the emission intensity therefore depend less on changes abroad and are mainly driven by national climate policies.

11 These categories are also in line with the household survey data in China Statistical Yearbook compiled by National Bureau of Statistics in China, which reflect the household consumption pattern each year. The expenditure of household on eight consumption categories are: food; clothing; housing; home equipment, facilities and services; health and medical care; transportation and communication; education, cultural and recreational services; and miscellaneous goods and services.

12 Further details are given in Appendix A. In particular Table A2 provides information at the product level, which suggests that the development of the digital economy in China might be one of the underlying reasons.

13 Household consumption shares in Bulgaria in 2011 on Clothing, Construction, Food, Manufacturing products, Mobility, Services and Shelter are 4%, 1%, 21%, 8%, 14%, 39%, and 13%, respectively.

14 Note that the emission multiplier of household consumption of a product is different from the traditional emission multiplier of producing a final product. Household consumption of a particular product is actually a composite of the same products sourced from different countries (including from the country in which the households consume).

15 Our results are consistent with findings by the World Bank. They predict an annual growth rate of world GDP of 2.81% from 2012 to 2018, ours is 2.42% for 2011–2030. Using model-based analyses and expert judgement, the OECD forecasts a global GDP growth of 3% during 2012–2030 (https://data.oecd.org/gdp/gdp-long-term-forecast.htm#indicator-chart).

## References

[1] IPCC (2023). https://www.ipcc.ch/assessment-report/ar6/.

[2] National Bureau of Statistics of China (2021).

[3] Duchin F., Lange G.M. (1994).

[4] Minx J.C., Baiocchi G., Peters G.P., Weber C.L., Guan D., Hubacek K. (2011). A “carbonizing dragon”: China's fast growing CO2 emissions revisited. Environ. Sci. Technol..

[5] Feng K., Hubacek K. (2016). Carbon implications of China's urbanization. Energy Ecol. Environ..

[6] Wiedenhofer D., Guan D., Liu Z., Meng J., Zhang N., Wei Y.M. (2017). Unequal household carbon footprints in China. Nat. Clim. Change.

[7] Ito K., Zhang S. (2016).

[8] Wong E. (2013). China, Breathing Becomes a Childhood Risk.

[9] NDRC (2022).

[10] Janssens-Maenhout G., Crippa M., Guizzardi D., Muntean M., Schaaf E. (2014). http://data.europa.eu/89h/jrc-edgar-jrc-edgar_v4-2_ft2010_timeseries.

[11] Guan D., Hubacek K., Weber C.L., Peters G.P., Reiner D.M. (2008). The drivers of Chinese CO 2 emissions from 1980 to 2030. Global Environ. Change.

[12] Hubacek K., Guan D., Barua A. (2007). Changing lifestyles and consumption patterns in developing countries: a scenario analysis for China and India. Futures.

[13] Hubacek K., Guan D., Barrett J., Wiedmann T. (2009). Environmental implications of urbanization and lifestyle change in China: ecological and water footprints. J. Clean. Prod..

[14] Wood R., Stadler K., Bulavskaya T., Lutter S., Giljum S., de Koning A., Kuenen J., Schütz H., Acosta-Fernández J., Usubiaga A., Simas M., Ivanova O., Weinzettel J., Schmidt J., Merciai S., Tukker A. (2015). Global sustainability accounting—developing EXIOBASE for multi-regional footprint analysis. Sustainability.

[15] WRAP Waste and resources action Programme. http://www.wrap.org.uk/.

[16] Gustavsson J., Cederberg C., Sonesson U., Van Otterdijk R., Meybeck A. (2011).

[17] Duan Y., Dietzenbacher E., Jiang X., Chen X., Yang C. (2018). Why has China's vertical specialization declined?. Econ. Syst. Res..

[18] Koopman R., Wang Z., Wei S.J. (2012). Estimating domestic content in exports when processing trade is pervasive. J. Dev. Econ..

[19] Kuznets S. (1955). Economic growth and income inequality. Am. Econ. Rev..

[20] Grossman G.M., Krueger A.B. (1995). Economic growth and the environment. Q. J. Econ..

[21] Dinda S. (2004). Environmental Kuznets curve hypothesis: a survey. Ecol. Econ..

[22] Suh S. (2004). Functions, commodities and environmental impacts in an ecological–economic model. Ecol. Econ..

[23] Hertwich E.G. (2005). Life cycle approaches to sustainable consumption: a critical review. Environ. Sci. Technol..

[24] Weber C.L., Matthews H.S. (2008). Quantifying the global and distributional aspects of American household carbon footprint. Ecol. Econ..

[25] Wiedmann T. (2009). A review of recent multi-region input–output models used for consumption-based emission and resource accounting. Ecol. Econ..

[26] Murray J., Wood R. (2010).

[27] Hertwich E.G., Peters G.P. (2009). Carbon footprint of nations: a global, trade-linked analysis. Environ. Sci. Technol..

[28] Ivanova D., Stadler K., Steen-Olsen K., Wood R., Vita G., Tukker A., Hertwich E.G. (2016). Environmental impact assessment of household consumption. J. Ind. Ecol..

[29] Sun M., Chen G., Xu X., Zhang L., Hubacek K., Wang Y. (2021). Reducing carbon footprint inequality of household consumption in rural areas: analysis from five representative provinces in China. Environ. Sci. Technol..

[30] Weber C., Perrels A. (2000). Modelling lifestyle effects on energy demand and related emissions. Energy Pol..

[31] De Koning A., Huppes G., Deetman S., Tukker A. (2016). Scenarios for a 2°C world: a trade-linked input–output model with high sector detail. Clim. Pol..

[32] Almon C. (1991). The INFORUM approach to interindustry modeling. Econ. Syst. Res..

[33] Meyer B., Lutz C., Schnur P., Zika G. (2007). National economic policy simulations with global interdependencies: a sensitivity analysis for Germany. Econ. Syst. Res..

[34] Dai H., Masui T., Matsuoka Y., Fujimori S. (2012). The impacts of China's household consumption expenditure patterns on energy demand and carbon emissions towards 2050. Energy Pol..

[35] Duarte R., Feng K., Hubacek K., Sánchez-Chóliz J., Sarasa C., Sun L. (2016). Modeling the carbon consequences of pro-environmental consumer behavior. Appl. Energy.

[36] Dietzenbacher E., Los B., Stehrer R., Timmer M., De Vries G. (2013). The construction of world input–output tables in the WIOD project. Econ. Syst. Res..

[37] Lenzen M., Kanemoto K., Moran D., Geschke A. (2012). Mapping the structure of the world economy. Environ. Sci. Technol..

[38] OECD (2015).

[39] Peters G.P., Davis S.J., Andrew R. (2012). A synthesis of carbon in international trade. Biogeosciences.

[40] Tukker A., Dietzenbacher E. (2013). Global Multiregional input–output Frameworks: an introduction and outlook. Econ. Syst. Res..

[41] Stadler K., Wood R., Bulavskaya T., Södersten C.J., Simas M., Schmidt S., Usubiaga A., Acosta-Fernández J., Kuenen J., Bruckner M., Giljum S., Lutter S., Merciai S., Schmidt J.H., Theurl M.C., Plutzar C., Kastner T., Eisenmenger N., Erb K.-H., de Koning A., Tukker A. (2018). Exiobase 3: developing a time series of detailed environmentally extended multi-regional input-output tables. J. Ind. Ecol..

[42] Boyce J.K., Gallagher K.P. (2008). Handbook on Trade and the Environment.

[43] Langdon D. (2009).

[44] Lundström R. (2017).

[45] Ouyang J., Hokao K. (2009). Energy-saving potential by improving occupants' behavior in urban residential sector in Hangzhou City, China. Energy Build..

[46] Peters G.P., Andrew R.M., Solomon S., Friedlingstein P. (2015). Measuring a fair and ambitious climate agreement using cumulative emissions. Environ. Res. Lett..

[47] Lenzen M., Keyer L., Hickel J. (2022). Degrowth scenarios for emissions neutrality. Nat. Food.

