# Supplementary material for: The global emission mitigation potential of avoiding waste and product lifespan extension by Chinese households
Source: Heliyon. 2024 Jan 23;10(3):e24322. doi: 10.1016/j.heliyon.2024.e24322 (PMC10843998; doi:10.1016/j.heliyon.2024.e24322)
Supplement: Multimedia component 1 [file mmc1.docx]

**Appendix A. The choice of Engel function specification**

Although several functional forms have been used in the estimation of Engel Curves, there is no agreement on a specific form [1]. It is assumed that a set of Engel functions (one for each product) can be derived from budget data, with the product’s expenditure share as the dependent variable and total expenditure as the independent variable. The Engel functions should have the same mathematical form for each product and should satisfy the additivity condition, which means that the expenditure shares for all products should add up to one. In this paper, we particularly investigate the following five functional forms, which are widely used in the literature [2, 3, 4, 5].^[[1]](#footnote-1)^ Among the first three Engel functions (with two parameters), Equation (A3) is recommended by Leser (1963) for its good fit to cross-sectional data[6]. Furthermore, since the three-parameter functions are expected to describe the variations in expenditure shares better, we also select two attractive functions to investigate: the Double Semi-Log function (Equation A4 as suggested by [7]) and the quadratic Engel curve (i.e. equation A5 as recommended by [8]).

$w_{i}=\alpha_{i}+\beta_{i}u+\varepsilon_{i}$ (A1)

$w_{i}=\alpha_{i}+\beta_{i}/u+\varepsilon_{i}$ (A2)

$w_{i}=\alpha_{i}+\beta_{i}\log u+\varepsilon_{i}$ (A3)

$w_{i}=\alpha_{i}+\beta_{i}u+\gamma_{i}\log u+\varepsilon_{i}$ (A4)

$w_{i}=\alpha_{i}+\beta_{i}\log u+\gamma_{i}{(\log u)}^{2}+\varepsilon_{i}$ (A5)

where $w_{i}$ is the expenditure share on product category $i$ and $u$ is the total expenditure. The good-of-fitness statistics (R^2^) of these five functions are summarized in Table A1. Pooled ordinary least squares regressions are used. Hence, each result is based on 833 observations (49 countries, 17 years).

As shown in Table A1, the fit of three-parameter functions (i.e. Equation (A4) and (A5)) is better than the two-parameter functions, in general. To choose between Equation (A4) and Equation (A5), we should take another criterion for selecting our preferred Engel curve specification into consideration. That is, the predicted expenditure shares for consumption categories should all be positive. According to Table A2, the predicted expenditure share for Services in 2011 obtained by using Equation (A4) is negative, which is sufficient to reject it. In contrast, Equation (A5) performs better for the predictions and should be selected for analysis. The third consideration in choosing an Engel function specification relates to having a close connection with a direct or indirect utility function [6]. Equation (A5) can be viewed as a utility-derived demand system with Price-Independent Generalized Logarithmic (PIGLOG) preferences [8]. Based on these considerations , we choose the functional form of Equation (A5) in this analysis.

Table A1 Proportion of variance explained by various Engel functions

| No. | Product | Category | (A1) | (A2) | (A3) | (A4） | (A5) |
| --- | --- | --- | --- | --- | --- | --- | --- |
| 1 | Paddy rice | Food | 0.026 | 0.302 | 0.148 | 0.040 | 0.287 |
| 2 | Wheat | Food | 0.037 | 0.132 | 0.102 | 0.033 | 0.123 |
| 3 | Cereal grains nec | Food | 0.095 | 0.497 | 0.319 | 0.006 | 0.426 |
| 4 | Vegetables, fruit, nuts | Food | 0.007 | 0.495 | 0.25 | 0.761 | 0.512 |
| 5 | Oil seeds | Food | 0.04 | 0.049 | 0.078 | 0.114 | 0.078 |
| 6 | Sugar cane, sugar beet | Food | 0.036 | 0.406 | 0.189 | 0.228 | 0.352 |
| 8 | Crops nec | Food | 0.052 | 0.163 | 0.167 | 0.660 | 0.180 |
| 11 | Poultry | Food | 0.088 | 0.245 | 0.208 | 0.052 | 0.226 |
| 12 | Meat animals nec | Food | 0.004 | 0.018 | 0 | 0.055 | 0.049 |
| 13 | Animal products nec | Food | 0.038 | 0.041 | 0.127 | 0.715 | 0.132 |
| 14 | Raw milk | Food | 0.047 | 0.202 | 0.18 | 0.204 | 0.212 |
| 19 | Fish and other fishing products; services incidental of fishing | Food | 0.024 | 0.25 | 0.138 | 0.615 | 0.222 |
| 43 | Products of meat cattle | Food | 0.021 | 0.022 | 0 | 0.349 | 0.096 |
| 44 | Products of meat pigs | Food | 0.058 | 0.021 | 0.012 | 0.111 | 0.213 |
| 45 | Products of meat poultry | Food | 0.044 | 0.019 | 0.009 | 0.164 | 0.167 |
| 46 | Meat products nec | Food | 0.001 | 0.041 | 0.013 | 0.204 | 0.069 |
| 47 | products of Vegetable oils and fats | Food | 0.07 | 0.193 | 0.207 | 0.232 | 0.224 |
| 48 | Dairy products | Food | 0.039 | 0.027 | 0.004 | 0.489 | 0.175 |
| 49 | Processed rice | Food | 0.038 | 0.078 | 0.109 | 0.050 | 0.115 |
| 50 | Sugar | Food | 0.117 | 0.044 | 0.149 | 0.065 | 0.187 |
| 51 | Food products nec | Food | 0.007 | 0.001 | 0.02 | 0.818 | 0.065 |
| 52 | Beverages | Food | 0.004 | 0.003 | 0.013 | 0.562 | 0.014 |
| 53 | Fish products | Food | 0.081 | 0.12 | 0.161 | 0.419 | 0.161 |
| 7 | Plant-based fibers | Clothing | 0.035 | 0.009 | 0.005 | 0.700 | 0.005 |
| 15 | Wool, silk-worm cocoons | Clothing | 0.015 | 0.002 | 0.017 | 0.010 | 0.026 |
| 55 | Textiles | Clothing | 0.005 | 0.129 | 0.064 | 0.802 | 0.113 |
| 56 | Wearing apparel; furs | Clothing | 0.045 | 0.001 | 0.027 | 0.673 | 0.079 |
| 57 | Leather and leather products | Clothing | 0.053 | 0.003 | 0.025 | 0.654 | 0.150 |
| 54 | Tobacco products | Manufactured products | 0.025 | 0.009 | 0.054 | 0.638 | 0.064 |
| 63 | Printed matter and recorded media | Manufactured products | 0.037 | 0.087 | 0.114 | 0.745 | 0.117 |
| 118 | Machinery and equipment n.e.c. | Manufactured products | 0.003 | 0.044 | 0.017 | 0.741 | 0.046 |
| 119 | Office machinery and computers | Manufactured products | 0.007 | 0 | 0 | 0.298 | 0.007 |
| 120 | Electrical machinery and apparatus n.e.c. | Manufactured products | 0.005 | 0.092 | 0.053 | 0.776 | 0.095 |
| 121 | Radio, television and communication equipment and apparatus | Manufactured products | 0.03 | 0.018 | 0.039 | 0.570 | 0.040 |
| 122 | Medical, precision and optical instruments, watches and clocks | Manufactured products | 0.006 | 0.051 | 0.047 | 0.477 | 0.059 |
| 124 | Other transport equipment | Manufactured products | 0.019 | 0.056 | 0.069 | 0.446 | 0.071 |
| 58 | Wood and products of wood and cork (except furniture); articles of straw and plaiting materials | Construction | 0.012 | 0.007 | 0.002 | 0.267 | 0.039 |
| 103 | Other non-metallic mineral products | Construction | 0.001 | 0.001 | 0.003 | 0.730 | 0.008 |
| 104 | Basic iron and steel and of ferro-alloys and first products thereof | Construction | 0 | 0.03 | 0.013 | 0.043 | 0.035 |
| 150 | Construction work | Construction | 0.005 | 0.006 | 0 | 0.155 | 0.025 |
| 123 | Motor vehicles, trailers and semi-trailers | Mobility | 0.007 | 0.057 | 0.044 | 0.576 | 0.056 |
| 152 | Sale, maintenance, repair of motor vehicles, motor vehicles parts, motorcycles, motor cycles parts and accessoiries | Mobility | 0.064 | 0.049 | 0.142 | 0.518 | 0.155 |
| 153 | Retail trade services of motor fuel | Mobility | 0 | 0.011 | 0.005 | 0.097 | 0.015 |
| 157 | Railway transportation services | Mobility | 0.004 | 0.011 | 0 | 0.671 | 0.052 |
| 158 | Other land transportation services | Mobility | 0.015 | 0.015 | 0.032 | 0.691 | 0.035 |
| 159 | Transportation services via pipelines | Mobility | 0.015 | 0.062 | 0.061 | 0.060 | 0.078 |
| 160 | Sea and coastal water transportation services | Mobility | 0.005 | 0.006 | 0.014 | 0.154 | 0.014 |
| 161 | Inland water transportation services | Mobility | 0.002 | 0.005 | 0.009 | 0.168 | 0.009 |
| 162 | Air transport services | Mobility | 0.004 | 0.008 | 0.012 | 0.428 | 0.012 |
| 18 | Products of forestry, logging and related services | Shelter | 0.051 | 0.157 | 0.134 | 0.163 | 0.148 |
| 62 | Paper and paper products | Shelter | 0.009 | 0.068 | 0.062 | 0.835 | 0.080 |
| 90 | Chemicals nec | Shelter | 0.013 | 0.033 | 0 | 0.861 | 0.124 |
| 96 | Rubber and plastic products | Shelter | 0.001 | 0.004 | 0.008 | 0.797 | 0.012 |
| 97 | Glass and glass products | Shelter | 0 | 0.001 | 0.002 | 0.059 | 0.002 |
| 117 | Fabricated metal products, except machinery and equipment | Shelter | 0.011 | 0.001 | 0.013 | 0.401 | 0.024 |
| 125 | Furniture; other manufactured goods n.e.c. | Shelter | 0.017 | 0.047 | 0.053 | 0.851 | 0.059 |
| 126 | Secondary raw materials | Shelter | 0 | 0.002 | 0.001 | 0.013 | 0.004 |
| 128 | Electricity by coal | Shelter | 0.007 | 0.007 | 0.006 | 0.151 | 0.007 |
| 129 | Electricity by gas | Shelter | 0.002 | 0 | 0.002 | 0.227 | 0.003 |
| 130 | Electricity by nuclear | Shelter | 0.001 | 0.017 | 0.003 | 0.114 | 0.030 |
| 131 | Electricity by hydro | Shelter | 0.004 | 0 | 0.003 | 0.178 | 0.007 |
| 132 | Electricity by wind | Shelter | 0.023 | 0.016 | 0.042 | 0.051 | 0.046 |
| 133 | Electricity by petroleum and other oil derivatives | Shelter | 0.016 | 0.001 | 0.003 | 0.036 | 0.035 |
| 134 | Electricity by biomass and waste | Shelter | 0.042 | 0.044 | 0.093 | 0.143 | 0.096 |
| 135 | Electricity by solar photovoltaic | Shelter | 0.002 | 0.005 | 0.008 | 0.026 | 0.008 |
| 136 | Electricity by solar thermal | Shelter | 0 | 0 | 0 | 0.004 | 0.001 |
| 137 | Electricity by tide, wave, ocean | Shelter | 0 | 0.001 | 0.001 | 0.011 | 0.002 |
| 138 | Electricity by Geothermal | Shelter | 0.036 | 0.043 | 0.088 | 0.014 | 0.089 |
| 139 | Electricity nec | Shelter | 0 | 0.016 | 0.006 | 0.083 | 0.028 |
| 140 | Transmission services of electricity | Shelter | 0.001 | 0.003 | 0 | 0.340 | 0.006 |
| 141 | Distribution and trade services of electricity | Shelter | 0 | 0.003 | 0.002 | 0.330 | 0.004 |
| 147 | Distribution services of gaseous fuels through mains | Shelter | 0.002 | 0.019 | 0.002 | 0.191 | 0.038 |
| 148 | Steam and hot water supply services | Shelter | 0.035 | 0.003 | 0.017 | 0.007 | 0.103 |
| 154 | Wholesale trade and commission trade services, except of motor vehicles and motorcycles | Services | 0.002 | 0.193 | 0.122 | 0.676 | 0.271 |
| 155 | Retail trade services, except of motor vehicles and motorcycles; repair services of personal and household goods | Services | 0.001 | 0.072 | 0.034 | 0.389 | 0.091 |
| 156 | Hotel and restaurant services | Services | 0.018 | 0.012 | 0.043 | 0.682 | 0.051 |
| 163 | Supporting and auxiliary transport services; travel agency services | Services | 0.084 | 0.062 | 0.094 | 0.732 | 0.094 |
| 164 | Post and telecommunication services | Services | 0.002 | 0.126 | 0.035 | 0.628 | 0.180 |
| 165 | Financial intermediation services, except insurance and pension funding services | Services | 0.016 | 0.032 | 0.046 | 0.473 | 0.046 |
| 166 | Insurance and pension funding services, except compulsory social security services | Services | 0.026 | 0.021 | 0.05 | 0.444 | 0.058 |
| 167 | Services auxiliary to financial intermediation | Services | 0.004 | 0.002 | 0.006 | 0.121 | 0.009 |
| 168 | Real estate services | Services | 0.161 | 0.216 | 0.355 | 0.815 | 0.357 |
| 169 | Renting services of machinery and equipment without operator and of personal and household goods | Services | 0 | 0.003 | 0 | 0.156 | 0.005 |
| 170 | Computer and related services | Services | 0.024 | 0.065 | 0.072 | 0.087 | 0.080 |
| 171 | Research and development services | Services | 0.007 | 0 | 0.005 | 0.017 | 0.017 |
| 172 | Other business services | Services | 0.014 | 0.015 | 0.013 | 0.775 | 0.014 |
| 173 | Public administration and defence services; compulsory social security services | Services | 0.001 | 0.032 | 0.008 | 0.201 | 0.045 |
| 174 | Education services | Services | 0.008 | 0.089 | 0.034 | 0.579 | 0.067 |
| 175 | Health and social work services | Services | 0.028 | 0.001 | 0.006 | 0.314 | 0.068 |
| 197 | Recreational, cultural and sporting services | Services | 0.003 | 0.076 | 0.06 | 0.562 | 0.087 |
| 198 | Other services | Services | 0 | 0.001 | 0.001 | 0.492 | 0.006 |
| 199 | Private households with employed persons | Services | 0 | 0 | 0 | 0.213 | 0.003 |

Note: the functions are estimated by using the data of consumption share and consumption level per capita for 49 countries over 1995 to 2011. -- indicate no household consumption data and thus no Engel function is estimated for the corresponding product.

As shown in Table A2, the predicted share of Clothing, Construction, and Manufacturing products are relatively close to the actual levels in 2011. However, the predicted share of Services is 7 percentage points lower than its actual value, while those for Food, Mobility and Shelter are 3 percentage points higher than the actual values. This reveals that Chinese households spend relatively much on Services and little on Food, Mobility and Shelter, compared to what would be expected on the basis of expenditure patterns of households in countries with similar standards of living. One possible reason is that with the development of digital economy in China, the share of online business and shopping gained in importance. This could be the reason of the high shares of expenditure on Services (such as *Post and telecommunication services (164), Financial intermediation services (165), Computer and related services (170)* etc.) in total household expenditure. These discrepancies between actual shares and predicted shares would imply a sudden change of the household consumption pattern from 2011 to 2012, if we would model household behavior entirely based on the estimated world Engel curves. To smooth the projected consumption path obtained from $\bar{w}_{1(t)i}^{s}$, we apply the following autoregressive projection. The household consumption pattern is assumed to be a combination of actual consumption share and prediction consumption share, with 0.5 and 0.5 as the weights:

$w_{1\left( 2012 \right)i}^{s}=0.5*w_{1\left( 2011 \right)i}^{s}+0.5*\bar{w}_{1\left( i,2012 \right)}^{s}$

$w_{1\left( i,t+1 \right)}^{s}=0.5*\bar{w}_{1\left( i,t \right)}^{s}+0.5*\bar{w}_{1\left( i,t+1 \right)}^{s}, for t\geq2012$

where$\bar{w}_{1\left( i,t \right)}^{s}$ is the predicted household consumption share for product $i$ in country $s$ in year $t$ according to the world Engel curves. As a robustness check, we also take 0.7 and 0.3, 0.9 and 0.1 as weights for the scenario analysis (See Appendix E).

Table A2 final demand structure of Chinese households (Unit: %)

|  | 2011 actual  (%) |  | 2011 predicted (%) | | | | |
| --- | --- | --- | --- | --- | --- | --- | --- |
|  |  |  | (A1) | (A2) | (A3) | (A4) | (A5) |
| Clothing | 6 |  | 5 | 5 | 5 | 11 | 5 |
| Construction | 1 |  | 1 | 1 | 1 | 1 | 1 |
| Food | 25 |  | 22 | 25 | 28 | 69 | 28 |
| Manufacturing products | 5 |  | 6 | 6 | 6 | 7 | 6 |
| Mobility | 7 |  | 11 | 10 | 10 | 7 | 10 |
| Services | 46 |  | 43 | 43 | 38 | -11 | 39 |
| Shelter | 9 |  | 12 | 11 | 12 | 16 | 12 |
| Total | 100 |  | 100 | 100 | 100 | 100 | 100 |

Note: Cells denote the share of a category in the total consumption expenditure.

**Appendix B. Details of the scenario model**

Focusing on the mathematical exposition of the scenario model, the rest of this section is organized as follows. Section B.1 elaborates on the final demand modules. The world input-output module and the emission module are described in Sections B.2 and section B.3, respectively. Section B.4 illustrates the scenario specifications.

**B.1 Final demand modules**

This section discusses how the final demand vectors are modeled. Product demand is given for household consumption (Section B.1.1) and for other final demand categories (Section B.1.2). After that the demand is split according to country of origin with the sourcing structure model (Section B.1.3).

B.1.1 Household consumption

The household final consumption model describes how household expenditures on each good and service vary with the income level. Based on the Absolute Income Hypothesis by [9], we model household consumption on a per capita basis in three steps. First, this year’s overall consumption per capita level depends on last year’s income per capita. Then, on the basis of this overall consumption level and the “world Engel curves”, the product-wise consumption shares per capita are obtained. Finally, the household consumption bundle is calculated.

The ratio between the total consumption level per capita in country $s$ in year $t$ (indicated by $u_{t}^{s}$) and the income level per capita in year $t-1$ is given by

$\lambda_{1(t)}^{s}=\frac{c_{t}^{s}/n_{t}^{s}}{v_{t-1}^{s}/n_{t-1}^{s}}=\frac{u_{t}^{s}}{v_{t-1}^{s}/n_{t-1}^{s}} (t=1996,\cdots,2011)$

with for country $s$ in year $t$, $c_{t}^{s}$ = total consumption level, $n_{t}^{s}$ = population,^[[2]](#footnote-2)^ $v_{t}^{s}$ = GDP (in constant prices), $u_{t}^{s}$ = consumption level per capita. It turns out that the ratio $\lambda_{1(t)}^{s}$ is fairly stable over time.^^[[3]](#footnote-3)^^ Therefore, we assume that this will also hold for the projection period, which implies that the income distribution does not change over time. For our projections we will use the average ratio $\lambda_{1}^{s}$, which is defined as

$\lambda_{1}^{s}=\frac{1}{16}\sum_{t=1996}^{2011} \lambda_{1(t)}^{s}$

The projection (indicated by an overbar) for the consumption per capita in year $t$ is obtained by multiplying GDP per capita in year $t-1$ with the average ratio $\lambda_{1}^{s}$. That is,

$\bar{u}_{t}^{s}=\lambda_{1}^{s}\frac{v_{t-1}^{s}}{n_{t-1}^{s}} (\mathrm{for} t=2012,\cdots2030)$ (B1)

Secondly, we determine projections for the consumption shares ${\bar{\mathbf{w}}}_{1(t)}^{s}$. The consumption shares change with total consumption expenditures. Their relationship is based on a series of product-specific Engel curves. The assumption is that these curves apply globally. This implies that if poor countries increase their average income, the consumption shares will change accordingly. Consumers will emulate the lifestyle of the rich when they get richer themselves [10], so the relationship between $\mathbf{w}_{1(t)}^{s}$ and $u_{t}^{s}$ (total consumption) is specified as

$\bar{w}_{1(t)i}^{s}=\hat{\alpha}_{i}+\hat{\beta}_{i}\log\bar{u}_{t}^{s}+\hat{\gamma}_{i}{(\log\bar{u}_{t}^{s})}^{2}$ $(\mathrm{for} t=2012,\cdots2030)$

with $\hat{\alpha}_{i}$, $\hat{\beta}_{i}$, and $\hat{\gamma}_{i}$ the OLS estimates (by using the data of consumption share^[[4]](#footnote-4)^ and total consumption level per capita in each country from 1995 to 2011) and $\bar{u}_{t}^{s}$ the projections obtained from (B1). Appendix A shows that this specification is a quadratic form of the Engel curve, which satisfies many desirable properties, including a larger good-of-fitness, positive predicted values and a better connection with the utility function [6].^[[5]](#footnote-5)^

As discussed in Appendix A already we apply the following autoregressive projection to smooth the projected consumption path obtained from $\bar{w}_{1(t)i}^{s}$:

${\bar{\bar{w}}}_{1(t)i}^{s}=r\bar{w}_{1(t-1)i}^{s}+(1-r)\bar{w}_{1(t)i}^{s} (\mathrm{for} t=2012,\cdots2030)$

where $r$ is the weight applied to the consumption share in year $t-1$. Please refer to Appendix D for the detailed discussions.

Finally, when the total consumption level and consumption structure are determined, the household consumption bundle of the whole economy in year $t$ can be determined. Recall that the $m$-element vectors of final demands are given by $\mathbf{f}_{k(t)}^{\cdot s}=\sum_{r} \mathbf{f}_{k(t)}^{rs}$, with *k* = 1, …, 4. The vector for household consumption is $\mathbf{f}_{1(t)}^{\cdot s}$, which gives, for each product separately, the total consumption in country *s*, i.e. irrespective of the origin of the product. Then the projections are obtained as

${\bar{\mathbf{f}}}_{1(t)}^{\cdot s}={\bar{\mathbf{w}}}_{1(t)}^{s}\bar{u}_{t}^{s}\bar{n}_{t}^{s} (\mathrm{for} t=2012,\cdots2030)$

B.1.2 Other final demands

Besides consumption, other final demands (${\bar{\mathbf{f}}}_{k(t)}^{\cdot s},k=2, 3, 4$) are the driving forces of growth in our model. Other final demands contain final consumption expenditures by non-profit organizations serving households (NPISH), government consumption, and gross fixed capital formation and inventory changes (GFCF). We first model the total level for each final demand category and then determine its composition in the second step. First, the total value of each final demand category is based on previous year’s GDP level. That is,

$\mathbf{1}'{\bar{\mathbf{f}}}_{k(t)}^{\cdot s}=\theta_{k}^{s}v_{t-1}^{s}$ (B2)

with $\mathbf{1}\mathbf{'}$ as the summation row vector and $\theta_{k}^{s}$ the consumption-to-GDP ratio, which is defined as the average of historical values. Second, the product-wise composition of each final demand, irrespective of the country from which sourcing takes place, is assumed to be fixed and the same as in the base year (2011). That is,

$w_{ik}^{s}=\frac{\sum_{r} f_{ik(2011)}^{rs}}{\sum_{i} \sum_{r} f_{ik(2011)}^{rs}}$ (B3)

When the total demand level and its corresponding structure are determined for each category, the levels of demand for each product in year $t$ can be determined as:

${\bar{\mathbf{f}}}_{k(t)}^{\cdot s}=\mathbf{w}_{k}^{s}\theta_{k}^{s}v_{t-1}^{s}$

With respect to NPISH (${\bar{\mathbf{f}}}_{2(t)}^{\cdot s}$), the main resources of non-profit organizations are voluntary contributions by households. Since their consumption level is related to previous year’s GDP level, (B2) applies. Considering that Non-profit institutions are mainly engaged in the production of non-market services for households [11] and emission multiplier for services are relatively small and similar, we assume (B3)

With respect to government consumption, $\mathbf{f}_{3(t)}$ presents government purchases of goods and services for current use. Therefore, it is highly related with the economy’s size. We thus model the government consumption expenditure as proportional to previous year’s GDP level, as in (B2). According to data in EXIOBASE’s WIOTs, government consumption is mainly concentrated in services, including defense, education, health, social security, public order and safety, general public expenditure. (B3) assumes the expenditure share of government spending to be constant

With respect to GFCF, this category shows how much of the production in an economy is used as a capital good rather than as a consumption good. The historical data from the WIOTs show that the GFCF-to-previous-year’s-GDP is relatively stable for most countries. Around 20%-25% for developed countries and higher ratios for emerging countries (for instance, the ratio in China was larger than 40% since 2004). Since business fluctuations are not considered in this analysis and the infrastructure in most emerging countries is still relatively weak, we model in (B2) the investment as proportional to previous year’s GDP level and the proportion is obtained as the average of the value (GFCF-to-previous-year’s-GDP) from 1996 to 2011.

B.1.3 Sourcing structure of final demands

Next, we incorporate the information of sourcing countries of final products into the obtained final demand patterns (${\bar{\mathbf{f}}}_{k(t)}^{\cdot s},k=1, 2, 3, 4$, with dimension $m\times1$, covering household consumption, NPISH, government consumption, and gross fixed capital formation and inventory changes) to arrive at the final demand vectors ${\bar{\mathbf{f}}}_{k\left( t \right)}^{s}(k=1, 2, 3, 4$, with dimension $mn\times1$). We first define the final demand trade structure vector (with dimension $mn\times1$), which answers the question where the final products come from:

$\mathbf{d}_{k(t)}^{s}=\left[ \begin{aligned} \mathbf{d}_{k(t)}^{1s} \\ \vdots\\ \mathbf{d}_{k(t)}^{ns} \end{aligned} \right],k=1, 2, 3, 4$

where $\mathbf{d}_{k(t)}^{rs}$ is of dimension $m\times1$ and its element $d_{ik}^{rs}=f_{ik}^{rs}/f_{ik}^{\cdot s}$ represents the share of final products $i$ (as required by final demand category $k$ in country $s$) that originates from country $r$. Note that $\sum_{r=1}^{n} d_{i1}^{rs}=1$ and $d_{i1}^{ss}$ in $\mathbf{d}_{1}^{ss}$shows the share of final products $i$ (required by household consumption in country $s$) originated from home country $s$. The assumptions of final demand trade structure vector $\mathbf{d}_{k}^{s}$ vary across scenarios: we assume it remains constant as in year 2011 in the scenarios BL and LC, while it changes in the trade pattern change scenarios (BLTP and LCTP). We will come back to this in Section B.4.2. Accordingly, we therefore have the final demand vectors ${\bar{\mathbf{f}}}_{k\left( t \right)}^{s}$ in year $t$ be determined as

${\bar{\mathbf{f}}}_{k(t)}^{s}=\left[ \begin{aligned} {\bar{\mathbf{d}}}_{k}^{1s}\circ{\bar{\mathbf{f}}}_{k(t)}^{\cdot s} \\ \vdots\\ {\bar{\mathbf{d}}}_{k}^{ns}\circ{\bar{\mathbf{f}}}_{k(t)}^{\cdot s} \end{aligned} \right],k=1, 2, 3, 4$

where $\circ$ indicates the Hadamard product of elementwise multiplication of matrices or vectors.

**B.2 Input-output module**

Input coefficients change over time for a variety of reasons, such as technological progress, substitution between domestic products and imported products, et cetera. Forecasting changes in input coefficients by taking into account all the driving factors is difficult. In our scenario analysis, we therefore only focus on one of the factors, trade structure. To this end, we first express the input coefficients $\mathbf{A}$ as the Hadamard product of the production technology $\mathbf{T}$ and intermediate trade structure $\mathbf{B}$, similar to the final demand. Define the $m\times m$ matrix $\mathbf{T}^{s}=\sum_{r=1}^{n} \mathbf{A}^{rs}$, which gives the product-wise intermediate inputs required by country $s$ per unit of production and thus represents the production technology in country $s$. Define $\mathbf{B}$ with its element $b_{ij}^{rs}=a_{ij}^{rs}/t_{ij}^{s}$, which reflects the share of intermediate inputs $i$ from country $r$ in the total inputs $i$ required per unit of production in product $j$ and country $s$. The matrix $\mathbf{B}$ ($mn\times mn$) can be expressed as

$\mathbf{B}=\left[ \begin{matrix} \mathbf{B}^{11} & \cdots& \mathbf{B}^{1n} \\ \vdots& \ddots& \vdots\\ \mathbf{B}^{n1} & \cdots& \mathbf{B}^{nn} \end{matrix} \right]$

In our scenario analysis, we assume $\mathbf{T}$ remains constant as in year 2011, that is $\mathbf{T}\mathbf{=}\mathbf{T}_{2011}$, considering that the production technology change is not the focus of this analysis. Moreover, the historical trend of the overall valued-added ratio in recent years is relatively stable. For example, Figure B1 shows that the value-added-to-output-ratio in China remained stable around 0.42 from 1995 to 2002. After that, the share of value-added in total output decreased sharply and became relatively stable after 2007 (around 0.35). The sharp decrease from 2002 to 2006 is possibly caused by the rise of processing trade in China after it entered WTO in 2001 [12]. At the sectoral level, the share of value added in total output is also found stable especially from 2007 to 2011.

For the trade structure of intermediate inputs $\mathbf{B}$, assumptions vary across scenarios: we assume it remains constant as in year 2011 in the baseline scenario, while changes in trade pattern change scenario. We will come back to this in Section B.4.2. Accordingly, the input coefficient matrix in year $t$ can be written as:

${\bar{\mathbf{A}}}_{t}=\left[ \begin{matrix} {\bar{\mathbf{B}}}_{t}^{11}\boldsymbol{\circ}\mathbf{T}^{1} & \boldsymbol{\cdots} & {\bar{\mathbf{B}}}_{t}^{1n}\boldsymbol{\circ}\mathbf{T}^{n} \\ \boldsymbol{\vdots} & \boldsymbol{\ddots} & \boldsymbol{\vdots} \\ {\bar{\mathbf{B}}}_{t}^{n1}\boldsymbol{\circ}\mathbf{T}^{1} & \boldsymbol{\cdots} & {\bar{\mathbf{B}}}_{t}^{nn}\boldsymbol{\circ}\mathbf{T}^{n} \end{matrix} \right]$

Figure B1. Share of total value-added in output in China during 1995-2011 (Source: EXIOBASE dataset)

**B.3 Emission module**

The EXIOBASE emission data include the major GHG emissions (CO_2_, CH_4_, N_2_O and SF_6_). We convert these GHG emissions to amounts of CO_2_-equivalent by multiplying the emissions with their Global Warming Potential with a time horizon of 100 years (as reported in IPCC 2013).^^[[6]](#footnote-6)^^ Consequently, the GHG emission coefficients at the industry level from 1995 to 2011 can be obtained by dividing the total amount of CO_2_-equivalent emissions by the output.

Considering that detailed modeling of emission intensity changes is not the main focus of the study, the emission coefficients are assumed to be constant for the projection period. As a robustness check, however, we also run the scenario analyses for the case where we have extrapolated the historical trend in emission coefficient changes of the top-5 emission-intensive products. These are: Cement, lime and plaster (industry 101); Electricity by coal (128); Electricity by gas (129); Electricity by petroleum and other oil derivatives (133); and Steam and hot water supply services (148). Their total emissions contributed to 35% of global emissions in 2011. The emission coefficients of other products are kept fixed.

In the literature on future carbon footprint scenarios, the treatment of future emission coefficients at the industry level can be classified into two categories. The first category models the future emission coefficients as a fraction of the original emission coefficients. For example, [13] assume that the emission coefficient of an industry in 2050 will be a factor 0.45 of the original coefficient. Instead of directly modeling the emission coefficient change, the second category models the energy use change in each industry, based on which the emissions at the industry level are calculated [14, 15]. The changes in the use of each energy carrier depend on each energy carrier’s production and relative energy prices, modeled by a CGE model [14] or an econometric input-output model [15]. The energy use changes are in turn affect any industry’s input coefficient for products from energy-related industries.

In our robustness checks, we also consider technological change. In this case, we assume that the annual “change factors” of the emission coefficients during the projection period are the same as the average annual change rate of the corresponding emission coefficient over the period 1995 to 2011. This method was used in [16] to project energy coefficients.^^[[7]](#footnote-7)^^ We do so for the top-5 emission-intensive products in the world.^[[8]](#footnote-8)^ The emission coefficients of other products are kept fixed. Expressed mathematically, if we denote the annual change rate of the emission coefficient of product $j$ in country $r$ as $\delta_{j}^{r}$, then the emission coefficient of product $j$ in country $r$ in year $t$ can be determined as

$\bar{\rho}_{(t)j}^{r}=\rho_{\left( 2011 \right)j}^{r}{(1+\delta_{j}^{r})}^{t-2011}$

which holds for $t=2012,\cdots2030$ and $j=101, 128, 129, 133, 148$. The results of this robustness analysis are summarized in Appendix F.

**B.4 Scenario description**

B.4.1 Low-carbon scenario

The scenarios we discussed in the main text, here we will only provide the technical details. The saved expenditures through reducing and changing consumption are re-spent by households on other products in the same category (on which savings are not possible). This re-spending is done in proportion to the existing composition (of the products that are involved). However, since household consumption of all the products classified in category of Clothing, Construction and Mobility can be saved, the expenditure savings cannot be re-spent in the same category. In this case, these expenditure savings are assumed to be re-spent in the “clean” category, that is, Services.^[[9]](#footnote-9)^

To mathematically express how the savings are re-spent, we first determine the total savings in each category and then deal with its composition in the second step. First, define the $m\times7$ savings matrix $\mathbf{Q}$ with element $q_{ij}$ indicating the possible saving rate of product $i$ that is classified in category $j$. Note that each product belongs to only one category. The projected $m\times1$ vector of Chinese household consumption in year $t$ in the baseline scenario (EM) is given by${\bar{\mathbf{f}}}_{1(t)}^{\cdot C}$. The 7$\times1$ vector with savings per category by Chinese households yields $\mathbf{Q}\mathbf{'}{\bar{\mathbf{f}}}_{1(t)}^{\cdot C}$. Note that the savings in the category Services are zero.

The categories Food, Manufactured products, and Shelter contain goods that can be saved and goods where savings are not possible. In the categories Clothing, Construction, and Mobility all goods can be saved. Nothing ca be saved in the category Services. Define an $m\times7$ indicator matrix $\mathbf{P}$ to indicate whether a product can be saved or not. That is, if the product $i$ in category $j$ cannot be saved, then $p_{ij}=1$, otherwise $p_{ij}=0$. Further, we define $p_{ij}=0$ if product $i$ does not belong to category $j$. The column for the category Food (and the same holds for Manufactured products and Shelter) contain ones only for the products in Food that cannot be saved. The savings in Food are then re-spent on the non-savable products in Food. The column for Clothing (and the same applies to Construction and Mobility) contains only zeroes. The column for Services contains ones for the all service products and zeroes for non-service products. The shares of the non-savable products (in Food, Manufactured products, and Shelter) and the service products are given by the $m\times7$ matrix $\mathbf{G}$. For $j$ corresponding to Food, Manufactured products, Shelter, and Services, element $g_{ij}={\bar{\mathbf{f}}}_{1(i,t)}^{\cdot C}\times p_{ij}/\sum_{i} ({\bar{\mathbf{f}}}_{1(i,t)}^{\cdot C}\times p_{ij})$. For $j$ corresponding to Clothing, Construction, or Mobility, the consumption structure of re-spending equals that for Services.

The$m$-element vector with extra demand for final products due to re-spending is given by $\mathbf{G} (\mathbf{Q}^{\mathbf{'}}{\bar{\mathbf{f}}}_{1\left( t \right)}^{\cdot C})$. Consequently, the household consumption patterns in China under low-carbon scenario ${\bar{\mathbf{f}}}_{1(t)}^{\cdot C,LC}$ consists of two parts. On the one hand, we have the reduction in household consumption due to low-carbon behavior, which equals ${\bar{\mathbf{f}}}_{1\left( t \right)}^{\cdot C}\circ\left( \mathbf{1}-\mathbf{Q1} \right)$. On the other hand, we have the extra consumption $\mathbf{G} (\mathbf{Q}^{\mathbf{'}}{\bar{\mathbf{f}}}_{1\left( t \right)}^{\cdot C})$ due to re-spending the savings from the low-carbon behavior. Taken together, this yields

${\bar{\mathbf{f}}}_{1(t)}^{\cdot C,LC}={\bar{\mathbf{f}}}_{1\left( t \right)}^{\cdot C}\circ\left( \mathbf{1}-\mathbf{Q1} \right)+\mathbf{G} (\mathbf{Q}^{\mathbf{'}}{\bar{\mathbf{f}}}_{1\left( t \right)}^{\cdot C})$

Once again, it should be stressed that shifting final demand to products with a lower global carbon footprint (such as substituting beef by pork) is not considered in the model. Therefore, this research evaluates the emission reduction by Chinese household through avoiding wastes and increasing the lifetime of products. This is a clear lower bound for the actual reduction that can be achieved.

B.4.2 Scenario with trade pattern changes

To check the robustness of our results, we also consider the case in which import shares for China decrease. The import shares of final demands decrease by 1% per year, those for the imports shares of intermediate inputs by 3% per year. For other countries, the trade structures of final demand and intermediate inputs are assumed to remain the same as in 2011.

China’s recent policy promotes domestic consumption. For the country origin of final demands, this implies that the share of domestic products will increase and the import share will decrease. According to the historical trend (Figure B2, based on the time series of WIOTs), the import share of Chinese final demands started to decrease after 2004 and increased slightly in 2010. As stated in the recent 13th five-year plan (2016-2020), the economic target is to increase average income and reduce inequality through restructuring the production system toward domestic demand and technological innovation. The slight increase in 2010 probably indicates a recovery after the financial crisis and the trend will decrease again afterwards.^^[[10]](#footnote-10)^^ The average annual change rate of imported final products share from 2004-2011 is 1%. We assume that this will continue during 2011-2030 (which implies that the import share will be 3.7% in 2030). That is, the final demand shares are

$\mathbf{d}_{k(t+1)}^{rC}=\left\{ \begin{aligned} \left( 1-0.01 \right)\mathbf{d}_{k\left( t \right)}^{rC},\mathrm{if} r\neq C,k=1,2,3,4 \\ 1-\sum_{r\neq C}^{n} \mathbf{d}_{k\left( t+1 \right)}^{rC}=\mathbf{d}_{k\left( t \right)}^{CC}+0.01\sum_{r\neq C}^{n} \mathbf{d}_{k\left( t \right)}^{rC},\mathrm{if} r=C,k=1,2,3,4 \end{aligned} \right.$

Figure B2. Share of imports in Chinese final demands during 2000-2011

For the sourcing structure of intermediate inputs in China, results suggesting a decrease in imported input coefficients was reported by [19] for 1997-2007 and by [12] for 2002-2012. This trend is expected to continue. First, because China’s policies advocate upgrading its position in global value chains. With economic development, China can now produce the imported components it could not produce before. Many firms thus shift the sourcing of inputs from foreign to domestic suppliers [20]. Second, with increasing labor costs the share of processing trade in total output will probably decrease in China. The typical feature is that production for processing trade depends much more on imported inputs than normal production. Less processing trade then leads to a smaller share of imported inputs in the total amount of intermediate inputs [21]. Figure B3 gives the share of imported inputs in the total amount of intermediate inputs and shows a consistent downward trend since 2004, with a disruption by the financial crisis in 2009. Extrapolation of the downward trend implies an annual 3% decrease (implying an import share of 6% in 2030). Then the input coefficients $\mathbf{B}_{t+1}^{rC}$ in China can be expressed as

$\mathbf{B}_{t+1}^{rC}=\left\{ \begin{aligned} \left( 1-0.03 \right)\mathbf{B}_{t}^{rC},\mathrm{if} r\neq C \\ \mathbf{B}_{t}^{CC}+0.03\sum_{r\neq C}^{n}\mathbf{B}_{t}^{rC},\mathrm{if} r=C \end{aligned} \right.$

Figure B3. The share of imported inputs in the total amount of intermediate inputs in China during 2000-2011

**Appendix C. Concordance between products and consumption categories**

The concordance table between products form EXIOBASE and the seven consumption categories

| No. | Product | Category | No. | Product | Category |
| --- | --- | --- | --- | --- | --- |
| 1 | Paddy rice | Food | 101 | Cement, lime and plaster | Construction |
| 2 | Wheat | Food | 102 | Ash for treatment, Re-processing of ash into clinker |  |
| 3 | Cereal grains nec | Food | 103 | Other non-metallic mineral products | Construction |
| 4 | Vegetables, fruit, nuts | Food | 104 | Basic iron and steel and of ferro-alloys and first products thereof | Construction |
| 5 | Oil seeds | Food | 105 | Secondary steel for treatment, Re-processing of secondary steel into new steel |  |
| 6 | Sugar cane, sugar beet |  | 106 | Precious metals |  |
| 7 | Plant-based fibers | Clothing | 107 | Secondary preciuos metals for treatment, Re-processing of secondary preciuos metals into new preciuos metals |  |
| 8 | Crops nec | Food | 108 | Aluminium and aluminium products |  |
| 9 | Cattle | Food | 109 | Secondary aluminium for treatment, Re-processing of secondary aluminium into new aluminium |  |
| 10 | Pigs | Food | 110 | Lead, zinc and tin and products thereof |  |
| 11 | Poultry | Food | 111 | Secondary lead for treatment, Re-processing of secondary lead into new lead |  |
| 12 | Meat animals nec | Food | 112 | Copper products |  |
| 13 | Animal products nec | Food | 113 | Secondary copper for treatment, Re-processing of secondary copper into new copper |  |
| 14 | Raw milk | Food | 114 | Other non-ferrous metal products | Construction |
| 15 | Wool, silk-worm cocoons | Clothing | 115 | Secondary other non-ferrous metals for treatment, Re-processing of secondary other non-ferrous metals into new other non-ferrous metals |  |
| 16 | Manure (conventional treatment) |  | 116 | Foundry work services |  |
| 17 | Manure (biogas treatment) |  | 117 | Fabricated metal products, except machinery and equipment | Shelter |
| 18 | Products of forestry, logging and related services | Shelter | 118 | Machinery and equipment n.e.c. | Manufactured products |
| 19 | Fish and other fishing products; services incidental of fishing | Food | 119 | Office machinery and computers | Manufactured products |
| 20 | Anthracite | Shelter | 120 | Electrical machinery and apparatus n.e.c. | Manufactured products |
| 21 | Coking Coal | Shelter | 121 | Radio, television and communication equipment and apparatus | Manufactured products |
| 22 | Other Bituminous Coal | Shelter | 122 | Medical, precision and optical instruments, watches and clocks | Manufactured products |
| 23 | Sub-Bituminous Coal | Shelter | 123 | Motor vehicles, trailers and semi-trailers | Mobility |
| 24 | Patent Fuel | Shelter | 124 | Other transport equipment | Manufactured products |
| 25 | Lignite/Brown Coal | Shelter | 125 | Furniture; other manufactured goods n.e.c. | Shelter |
| 26 | BKB/Peat Briquettes | Shelter | 126 | Secondary raw materials |  |
| 27 | Peat | Shelter | 127 | Bottles for treatment, Recycling of bottles by direct reuse |  |
| 28 | Crude petroleum and services related to crude oil extraction, excluding surveying |  | 128 | Electricity by coal | Shelter |
| 29 | Natural gas and services related to natural gas extraction, excluding surveying | Shelter | 129 | Electricity by gas | Shelter |
| 30 | Natural Gas Liquids |  | 130 | Electricity by nuclear | Shelter |
| 31 | Other Hydrocarbons |  | 131 | Electricity by hydro | Shelter |
| 32 | Uranium and thorium ores |  | 132 | Electricity by wind | Shelter |
| 33 | Iron ores |  | 133 | Electricity by petroleum and other oil derivatives | Shelter |
| 34 | Copper ores and concentrates |  | 134 | Electricity by biomass and waste | Shelter |
| 35 | Nickel ores and concentrates |  | 135 | Electricity by solar photovoltaic | Shelter |
| 36 | Aluminium ores and concentrates |  | 136 | Electricity by solar thermal | Shelter |
| 37 | Precious metal ores and concentrates |  | 137 | Electricity by tide, wave, ocean | Shelter |
| 38 | Lead, zinc and tin ores and concentrates |  | 138 | Electricity by Geothermal | Shelter |
| 39 | Other non-ferrous metal ores and concentrates |  | 139 | Electricity nec | Shelter |
| 40 | Stone | Construction | 140 | Transmission services of electricity | Shelter |
| 41 | Sand and clay | Construction | 141 | Distribution and trade services of electricity | Shelter |
| 42 | Chemical and fertilizer minerals, salt and other mining and quarrying products n.e.c. | Shelter | 142 | Coke oven gas |  |
| 43 | Products of meat cattle | Food | 143 | Blast Furnace Gas |  |
| 44 | Products of meat pigs | Food | 144 | Oxygen Steel Furnace Gas |  |
| 45 | Products of meat poultry | Food | 145 | Gas Works Gas |  |
| 46 | Meat products nec | Food | 146 | Biogas |  |
| 47 | products of Vegetable oils and fats | Food | 147 | Distribution services of gaseous fuels through mains | Shelter |
| 48 | Dairy products | Food | 148 | Steam and hot water supply services | Shelter |
| 49 | Processed rice | Food | 149 | Collected and purified water, distribution services of water | Shelter |
| 50 | Sugar | Food | 150 | Construction work | Construction |
| 51 | Food products nec | Food | 151 | Secondary construction material for treatment, Re-processing of secondary construction material into aggregates |  |
| 52 | Beverages | Food | 152 | Sale, maintenance, repair of motor vehicles, motor vehicles parts, motorcycles, motor cycles parts and accessoiries | Mobility |
| 53 | Fish products | Food | 153 | Retail trade services of motor fuel | Mobility |
| 54 | Tobacco products | Manufactured products | 154 | Wholesale trade and commission trade services, except of motor vehicles and motorcycles | Services |
| 55 | Textiles | Clothing | 155 | Retail trade services, except of motor vehicles and motorcycles; repair services of personal and household goods | Services |
| 56 | Wearing apparel; furs | Clothing | 156 | Hotel and restaurant services | Services |
| 57 | Leather and leather products | Clothing | 157 | Railway transportation services | Mobility |
| 58 | Wood and products of wood and cork (except furniture); articles of straw and plaiting materials | Construction | 158 | Other land transportation services | Mobility |
| 59 | Wood material for treatment, Re-processing of secondary wood material into new wood material |  | 159 | Transportation services via pipelines |  |
| 60 | Pulp |  | 160 | Sea and coastal water transportation services | Mobility |
| 61 | Secondary paper for treatment, Re-processing of secondary paper into new pulp |  | 161 | Inland water transportation services | Mobility |
| 62 | Paper and paper products | Shelter | 162 | Air transport services | Mobility |
| 63 | Printed matter and recorded media | Manufactured products | 163 | Supporting and auxiliary transport services; travel agency services | Services |
| 64 | Coke Oven Coke | Shelter | 164 | Post and telecommunication services | Services |
| 65 | Gas Coke |  | 165 | Financial intermediation services, except insurance and pension funding services | Services |
| 66 | Coal Tar |  | 166 | Insurance and pension funding services, except compulsory social security services | Services |
| 67 | Motor Gasoline | Mobility | 167 | Services auxiliary to financial intermediation | Services |
| 68 | Aviation Gasoline |  | 168 | Real estate services | Services |
| 69 | Gasoline Type Jet Fuel |  | 169 | Renting services of machinery and equipment without operator and of personal and household goods | Services |
| 70 | Kerosene Type Jet Fuel | Mobility | 170 | Computer and related services | Services |
| 71 | Kerosene | Shelter | 171 | Research and development services | Services |
| 72 | Gas/Diesel Oil | Mobility | 172 | Other business services | Services |
| 73 | Heavy Fuel Oil | Mobility | 173 | Public administration and defence services; compulsory social security services | Services |
| 74 | Refinery Gas |  | 174 | Education services | Services |
| 75 | Liquefied Petroleum Gases (LPG) | Shelter | 175 | Health and social work services | Services |
| 76 | Refinery Feedstocks |  | 176 | Food waste for treatment: incineration |  |
| 77 | Ethane |  | 177 | Paper waste for treatment: incineration | Shelter |
| 78 | Naphtha | Shelter | 178 | Plastic waste for treatment: incineration |  |
| 79 | White Spirit & SBP |  | 179 | Intert/metal waste for treatment: incineration | Shelter |
| 80 | Lubricants |  | 180 | Textiles waste for treatment: incineration |  |
| 81 | Bitumen |  | 181 | Wood waste for treatment: incineration |  |
| 82 | Paraffin Waxes |  | 182 | Oil/hazardous waste for treatment: incineration |  |
| 83 | Petroleum Coke |  | 183 | Food waste for treatment: biogasification and land application |  |
| 84 | Non-specified Petroleum Products | Shelter | 184 | Paper waste for treatment: biogasification and land application | Shelter |
| 85 | Nuclear fuel |  | 185 | Sewage sludge for treatment: biogasification and land application | Shelter |
| 86 | Plastics, basic |  | 186 | Food waste for treatment: composting and land application |  |
| 87 | Secondary plastic for treatment, Re-processing of secondary plastic into new plastic |  | 187 | Paper and wood waste for treatment: composting and land application |  |
| 88 | N-fertiliser | Shelter | 188 | Food waste for treatment: waste water treatment | Shelter |
| 89 | P- and other fertiliser | Shelter | 189 | Other waste for treatment: waste water treatment | Shelter |
| 90 | Chemicals nec | Shelter | 190 | Food waste for treatment: landfill | Shelter |
| 91 | Charcoal | Shelter | 191 | Paper for treatment: landfill | Shelter |
| 92 | Additives/Blending Components |  | 192 | Plastic waste for treatment: landfill | Shelter |
| 93 | Biogasoline | Mobility | 193 | Inert/metal/hazardous waste for treatment: landfill | Shelter |
| 94 | Biodiesels | Mobility | 194 | Textiles waste for treatment: landfill |  |
| 95 | Other Liquid Biofuels | Mobility | 195 | Wood waste for treatment: landfill |  |
| 96 | Rubber and plastic products | Shelter | 196 | Membership organisation services n.e.c. | Services |
| 97 | Glass and glass products |  | 197 | Recreational, cultural and sporting services | Services |
| 98 | Secondary glass for treatment, Re-processing of secondary glass into new glass |  | 198 | Other services | Services |
| 99 | Ceramic goods | Shelter | 199 | Private households with employed persons | Services |
| 100 | Bricks, tiles and construction products, in baked clay | Construction | 200 | Extra-territorial organizations and bodies |  |

Note: if the product is not demanded by households, then it is not assigned to any category.

**Appendix D. Product shares within categories**

Table 4 in the main text gave the household consumption shares for the seven categories, i.e. Clothing, Construction, Food, Manufacturing products, Mobility, Services, and Shelter. Table D1 gives the household consumption shares of the products within each category. Note that the product shares add to one for each category.

Table D1 Household consumption shares for products within each category in 2011 and in 2030 (under the BL scenario)

| Clothing | | |  | Food | | |
| --- | --- | --- | --- | --- | --- | --- |
|  | 2011 | BL |  |  | 2011 | BL |
| Textiles | 0.03 | 0.29 |  | Cereal grains nec | 0.03 | 0.04 |
| Wearing apparel; furs | 0.78 | 0.50 |  | Vegetables, fruit, nuts | 0.23 | 0.10 |
| Leather and leather products | 0.19 | 0.21 |  | Sugar cane, sugar beet | 0.00 | 0.01 |
|  |  |  |  | Crops nec | 0.00 | 0.03 |
| Construction | | |  | Poultry | 0.03 | 0.02 |
|  | 2011 | BL |  | Animal products nec | 0.00 | 0.02 |
| Wood and products of wood (except furniture) | 0.00 | 0.21 |  | Raw milk | 0.01 | 0.03 |
| Bricks, tiles and construction products | 0.00 | 0.00 |  | Fish and other fishing products; services incidental of fishing | 0.02 | 0.02 |
| Cement, lime and plaster | 0.00 | 0.01 |  | Products of meat cattle | 0.00 | 0.03 |
| Other non-metallic mineral products | 0.04 | 0.24 |  | Products of meat pigs | 0.01 | 0.04 |
| Construction work | 0.95 | 0.53 |  | Products of meat poultry | 0.01 | 0.05 |
|  |  |  |  | Meat products nec | 0.01 | 0.04 |
| Manufactured products | | |  | Products of vegetable oils and fats | 0.00 | 0.01 |
|  | 2011 | BL |  | Dairy products | 0.01 | 0.09 |
| Tobacco products | 0.08 | 0.19 |  | Processed rice | 0.02 | 0.02 |
| Printed matter and recorded media | 0.07 | 0.20 |  | Sugar | 0.00 | 0.02 |
| Machinery and equipment n.e.c. | 0.01 | 0.17 |  | Food products nec | 0.33 | 0.28 |
| Office machinery and computers | 0.08 | 0.05 |  | Beverages | 0.04 | 0.06 |
| Electrical machinery and apparatus n.e.c. | 0.46 | 0.07 |  | Fish products | 0.26 | 0.08 |
| Radio, television and communication equipment and apparatus | 0.21 | 0.20 |  |  |  |  |
| Medical, precision and optical instruments, watches and clocks | 0.04 | 0.07 |  | Shelter | | |
| Other transport equipment | 0.04 | 0.05 |  |  | 2011 | BL |
|  |  |  |  | Products of forestry, logging and related services | 0.04 | 0.03 |
| Mobility | | |  | Other Bituminous Coal | 0.01 | 0.01 |
|  | 2011 | BL |  | Paper and paper products | 0.01 | 0.04 |
| Motor Gasoline | 0.14 | 0.16 |  | Kerosene | 0.00 | 0.02 |
| Gas/Diesel Oil | 0.03 | 0.05 |  | Liquefied Petroleum Gases (LPG) | 0.00 | 0.01 |
| Motor vehicles, trailers and semi-trailers | 0.50 | 0.29 |  | Chemicals nec | 0.17 | 0.23 |
| Sale, maintenance, repair of motor vehicles, motor vehicles parts, motorcycles, motor cycles parts and accessoiries | 0.11 | 0.10 |  | Rubber and plastic products | 0.07 | 0.06 |
| Retail trade services of motor fuel | 0.01 | 0.01 |  | Ceramic goods | 0.01 | 0.02 |
| Railway transportation services | 0.08 | 0.11 |  | Fabricated metal products, except machinery and equipment | 0.03 | 0.07 |
| Other land transportation services | 0.05 | 0.19 |  | Furniture; other manufactured goods n.e.c. | 0.28 | 0.16 |
| Sea and coastal water transportation services | 0.01 | 0.01 |  | Electricity by coal | 0.08 | 0.02 |
| Air transport services | 0.05 | 0.08 |  | Electricity by gas | 0.00 | 0.01 |
|  |  |  |  | Electricity by nuclear | 0.00 | 0.02 |
| Services | | |  | Electricity by hydro | 0.02 | 0.02 |
|  | 2011 | BL |  | Electricity by petroleum and other oil derivatives | 0.01 | 0.01 |
| Wholesale trade and commission trade services, except of motor vehicles | 0.04 | 0.01 |  | Transmission services of electricity | 0.02 | 0.01 |
| Retail trade services, except of motor vehicles and motorcycles; repair services of personal and household goods | 0.05 | 0.02 |  | Distribution and trade services of electricity | 0.08 | 0.06 |
| Hotel and restaurant services | 0.12 | 0.17 |  | Distribution services of gaseous fuels through mains | 0.06 | 0.06 |
| Supporting and auxiliary transport services; travel agency services | 0.01 | 0.03 |  | Steam and hot water supply services | 0.00 | 0.03 |
| Post and telecommunication services | 0.00 | 0.08 |  | Collected and purified water, distribution services of water | 0.07 | 0.03 |
| Financial intermediation services, except insurance and pension funding services | 0.01 | 0.05 |  | Food waste for treatment: waste water treatment | 0.00 | 0.01 |
| Insurance and pension funding services, except compulsory social security services | 0.10 | 0.05 |  | Other waste for treatment: waste water treatment | 0.01 | 0.02 |
| Services auxiliary to financial intermediation | 0.00 | 0.01 |  | Food waste for treatment: landfill | 0.01 | 0.02 |
| Real estate services | 0.25 | 0.32 |  | Paper for treatment: landfill | 0.00 | 0.01 |
| Renting services of machinery and equipment without operator and of personal and household goods | 0.01 | 0.02 |  | Inert/metal/hazardous waste for treatment: landfill | 0.00 | 0.01 |
| Computer and related services | 0.08 | 0.01 |  |  |  |  |
| Other business services | 0.02 | 0.02 |  |  |  |  |
| Education services | 0.05 | 0.04 |  |  |  |  |
| Health and social work services | 0.13 | 0.07 |  |  |  |  |
| Recreational, cultural and sporting services | 0.02 | 0.06 |  |  |  |  |
| Other services | 0.08 | 0.04 |  |  |  |  |
| Private households with employed persons | 0.02 | 0.01 |  |  |  |  |

**Appendix E**

**r=0.7:**

Table E1 final demand structure of Chinese households (Unit: Billion euros)

|  | 2011 | |  | 2030 | | | | |  | Low-carbon effect 2030 | | | | |
| --- | --- | --- | --- | --- | --- | --- | --- | --- | --- | --- | --- | --- | --- | --- |
|  |  |  |  | BL | |  | LC | |  | LC minus BL | |  | LCTP minus BLTP | |
|  |  | % |  |  | % |  |  | % |  |  | % |  |  | % |
| Clothing | 93 | 6 |  | 204 | 5 |  | 146 | 3 |  | -58 | -1 |  | -80 | -1 |
| Construction | 18 | 1 |  | 49 | 1 |  | 40 | 1 |  | -9 | 0 |  | -13 | 0 |
| Food | 377 | 25 |  | 964 | 23 |  | 982 | 23 |  | 18 | 0 |  | 20 | 0 |
| Manufacturing products | 80 | 5 |  | 264 | 6 |  | 270 | 6 |  | 6 | 0 |  | 8 | 0 |
| Mobility | 105 | 7 |  | 466 | 11 |  | 239 | 5 |  | -227 | -5 |  | -327 | -6 |
| Services | 690 | 46 |  | 1781 | 42 |  | 2138 | 49 |  | 357 | 7 |  | 498 | 7 |
| Shelter | 135 | 9 |  | 535 | 13 |  | 548 | 13 |  | 13 | 0 |  | 15 | 0 |
| Total | 1498 | 100 |  | 4263 | 100 |  | 4363 | 100 |  | 100 | 0 |  | 121 | 0 |

Note: Columns with % represent the share of each category in the total consumption expenditure.

Table E2 Global footprint of Chinese household (Unit: Gt CO_2_-equivalent)

|  |  | |  |  | |  | Low-carbon effect | | | | |
| --- | --- | --- | --- | --- | --- | --- | --- | --- | --- | --- | --- |
|  | BL | |  | _LC | |  | LC minus BL | |  | LCTP minus BLTP | |
|  |  | % |  |  | % |  |  | % |  |  | % |
| Clothing | 0.31 | 3 |  | 0.22 | 2 |  | -0.09 | -1 |  | -0.13 | -1 |
| Construction | 0.18 | 2 |  | 0.14 | 1 |  | -0.03 | 0 |  | -0.05 | 0 |
| Food | 2.96 | 28 |  | 2.98 | 30 |  | 0.02 | 2 |  | -0.01 | 1 |
| Manufacturing products | 0.42 | 4 |  | 0.45 | 4 |  | 0.03 | 0 |  | 0.03 | 0 |
| Mobility | 0.83 | 8 |  | 0.43 | 4 |  | -0.41 | -4 |  | -0.61 | -4 |
| Services | 1.23 | 12 |  | 1.48 | 15 |  | 0.25 | 3 |  | 0.36 | 3 |
| Shelter | 4.63 | 44 |  | 4.37 | 43 |  | -0.26 | 0 |  | -0.39 | 0 |
| Total | 10.55 | 100 |  | 10.06 | 100 |  | -0.49 | 0 |  | -0.79 | 0 |

Note: Columns with % represents the shares of footprint by each category in the total consumption footprint.

Table E3 Projected global GDP and GHG emissions under different scenarios

|  |  |  |  |  | Low-carbon effect | |
| --- | --- | --- | --- | --- | --- | --- |
|  |  | BL | LC |  | LC minus BL | LCTP minus BLTP |
| GDP (B.Euros) | 2011 | 52304 | |  |  |  |
|  | 2030 | 82500 | 82735 |  | 0.5% | 0.5% |
| GHG emissions (Gt) | 2011 | 40.1 | |  |  |  |
|  | 2030 | 76.6 | 76.4 |  | -0.5% | -1.0% |

**r=0.9**

Table E4 final demand structure of Chinese households (Unit: Billion euros)

|  | 2011 | |  | 2030 | | | | |  | Low-carbon effect 2030 | | | | |
| --- | --- | --- | --- | --- | --- | --- | --- | --- | --- | --- | --- | --- | --- | --- |
|  |  |  |  | BL | |  | LC | |  | LC minus BL | |  | LCTP minus BLTP | |
|  |  | % |  |  | % |  |  | % |  |  | % |  |  | % |
| Clothing | 93 | 6 |  | 214 | 5 |  | 153 | 3 |  | -61 | -2 |  | -85 | -1 |
| Construction | 18 | 1 |  | 49 | 1 |  | 40 | 1 |  | -9 | 0 |  | -13 | 0 |
| Food | 377 | 25 |  | 1008 | 23.5 |  | 1026 | 23 |  | 18 | 0 |  | 21 | 0 |
| Manufacturing products | 80 | 5 |  | 260 | 6 |  | 299 | 7 |  | 6 | 0 |  | 7 | 0 |
| Mobility | 105 | 7 |  | 442 | 10 |  | 226 | 5 |  | -216 | -5 |  | -308 | -5 |
| Services | 690 | 46 |  | 1803 | 42 |  | 2144 | 49 |  | 342 | 7 |  | 474 | 7 |
| Shelter | 135 | 9 |  | 517 | 12 |  | 494 | 11 |  | 11 | 0 |  | 13 | 0 |
| Total | 1498 | 100 |  | 4293 | 100 |  | 4382 | 100 |  | 91 | 0 |  | 109 | 0 |

Note: Columns with % represent the share of each category in the total consumption expenditure.

Table E5 Global footprint of Chinese household (Unit: Gt CO_2_-equivalent)

|  |  | |  |  | |  | Low-carbon effect | | | | |
| --- | --- | --- | --- | --- | --- | --- | --- | --- | --- | --- | --- |
|  | BL | |  | LC | |  | LC minus BL | |  | LCTP minus BLTP | |
|  |  | % |  |  | % |  |  | % |  |  | % |
| Clothing | 0.32 | 3 |  | 0.23 | 2 |  | -0.09 | -1 |  | -0.13 | -1 |
| Construction | 0.17 | 2 |  | 0.14 | 1 |  | -0.03 | 0 |  | -0.05 | 0 |
| Food | 2.94 | 29 |  | 2.97 | 31 |  | 0.04 | 2 |  | 0.03 | 2 |
| Manufacturing products | 0.41 | 4 |  | 0.44 | 5 |  | 0.03 | 0 |  | 0.03 | 0 |
| Mobility | 0.79 | 8 |  | 0.40 | 4 |  | -0.38 | -4 |  | -0.58 | -4 |
| Services | 1.25 | 12 |  | 1.49 | 15 |  | 0.24 | 3 |  | 0.35 | 3 |
| Shelter | 4.31 | 42 |  | 4.07 | 42 |  | -0.24 | -1 |  | -0.35 | 0 |
| Total | 10.19 | 100 |  | 9.75 | 100 |  | -0.44 | 0 |  | -0.70 | 0 |

Note: Columns with % represents the shares of footprint by each category in the total consumption footprint.

Table E6 Projected global GDP and GHG emissions under different scenarios

|  |  |  |  |  | Low-carbon effect | |
| --- | --- | --- | --- | --- | --- | --- |
|  |  | BL | LC |  | LC minus BL | LCTP minus BLTP |
| GDP (B.Euros) | 2011 | 52304 | |  |  |  |
|  | 2030 | 82776 | 82985 |  | 0.4% | 0.4% |
| GHG emissions (Gt) | 2011 | 40.1 | |  |  |  |
|  | 2030 | 76.1 | 75.9 |  | -0.5% | -1.0% |

**Appendix F**

Table F1 final demand structure of Chinese households (Unit: Billion euros)

|  | 2011 | |  | 2030 | | | | |  | Low-carbon effect 2030 | | | | |
| --- | --- | --- | --- | --- | --- | --- | --- | --- | --- | --- | --- | --- | --- | --- |
|  |  |  |  | BL | |  | LC | |  | LC minus BL | |  | LCTP minus BLTP | |
|  |  | % |  |  | % |  |  | % |  |  | % |  |  | % |
| Clothing | 93 | 6 |  | 203 | 5 |  | 145 | 3 |  | -58 | -1 |  | -80 | -1 |
| Construction | 18 | 1 |  | 49 | 1 |  | 41 | 1 |  | -9 | 0 |  | -13 | 0 |
| Food | 377 | 25 |  | 955 | 22 |  | 972 | 22 |  | 18 | 0 |  | 20 | 0 |
| Manufacturing products | 80 | 5 |  | 264 | 6 |  | 270 | 6 |  | 6 | 0 |  | 8 | 0 |
| Mobility | 105 | 7 |  | 466 | 11 |  | 239 | 5 |  | -227 | -5 |  | -328 | -6 |
| Services | 690 | 46 |  | 1783 | 42 |  | 2142 | 49 |  | 358 | 7 |  | 501 | 7 |
| Shelter | 135 | 9 |  | 535 | 13 |  | 547 | 13 |  | 13 | 0 |  | 15 | 0 |
| Total | 1498 | 100 |  | 4255 | 100 |  | 4356 | 100 |  | 101 | 0 |  | 123 | 0 |

Note: Columns with % represent the share of each category in the total consumption expenditure.

Table F2 Global footprint of Chinese household when energy efficiency improvement is considered (Unit: Gt CO_2_-equivalent)

|  |  | |  |  | |  | Low-carbon effect | | | | |
| --- | --- | --- | --- | --- | --- | --- | --- | --- | --- | --- | --- |
|  | BL | |  | LC | |  | LC minus BL | |  | LCTP minus BLTP | |
|  |  | % |  |  | % |  |  | % |  |  | % |
| Clothing | 0.26 | 3 |  | 0.18 | 2 | 0.07 | | -1 |  | -0.11 | -1 |
| Construction | 0.12 | 1 |  | 0.10 | 1 |  | 0.02 | 0 |  | -0.03 | 0 |
| Food | 2.80 | 30 |  | 2.81 | 31 |  | -0.02 | 2 |  | -0.01 | 1 |
| Manufacturing products | 0.36 | 4 |  | 0.39 | 4 |  | -0.03 | 0 |  | 0.03 | 0 |
| Mobility | 0.75 | 8 |  | 0.39 | 4 |  | 0.37 | -4 |  | -0.55 | -4 |
| Services | 1.06 | 11 |  | 1.27 | 14 |  | -0.21 | 3 |  | 0.31 | 3 |
| Shelter | 4.05 | 43 |  | 3.83 | 43 |  | 0.22 | 0 |  | -0.33 | 0 |
| Total | 9.40 | 100 |  | 8.97 | 100 |  | 0.43 | 0 |  | -0.69 | 0 |

Note: Columns with % represents the shares of footprint by each category in the total consumption footprint.

Table F3 Projected global GDP and GHG emissions under different scenarios

|  |  |  |  |  | Low-carbon effect | |
| --- | --- | --- | --- | --- | --- | --- |
|  |  | BL | LC |  | LC minus BL | LCTP minus BLTP |
| GDP (B.Euros) | 2011 | 52304 | |  |  |  |
|  | 2030 | 82429 | 82672 |  | 0.5% | 0.5% |
| GHG emissions (Gt) | 2011 | 40.1 | |  |  |  |
|  | 2030 | 67.9 | 67.7 |  | -0.5% | -1.0% |

With considering energy efficiency improvement, global CO2 equivalent emissions will decrease in all scenarios as compared to those without energy efficiency improvements.

**References to appendices**

1. Abdel-Ghany, M., & Schwenk, F.N. (1993). Functional forms of household expenditure patterns in the United States. Journal of Consumer Studies and Home Economics, 17, 325-342.
2. Houthakker, H.S. (1957). An international comparison of household expenditure patterns commemorating the centennial of Engel’s Law. Econometrica, 25(4), 532–551.
3. Deaton, A., & Muellbauer, J. (1980). An almost ideal demand system. American Economic Review, 70, 312-326
4. Battese, G., & Bonyhady, B. (1981). Estimation of household expenditure functions: An application of a class of heteroscedastic regression models. Economic Record, 57(1), 80-85.
5. Wakabayashi, M., & Hewings, G. (2007). Life-cycle changes in consumption behavior: Age-specific and regional variations, Journal of Regional Science, 47(2), 315-337.
6. Leser, C. (1963). Forms of Engel functions. Econometrica, 31(4), 694-703.
7. Marquez, J., Adams, F.G., Balestra, P., Dagnenais, M.G., Kendrick, D., Paelinck, J.H.P., Pindyck, R.S., & Welfe, W. (2005). Functional forms for Engel curves. In: Marquez, J. et al. (eds) Income Elasticity and Economic Development. Advanced Studies in Theoretical and Applied Econometrics, 42. Springer, Boston, MA. https://doi.org/10.1007/0-387-24344-5_3.
8. Banks, J., Blundell, R., & Lewbel, A. (1997). Quadratic Engel curves and consumer demand. Review of Economic Statistics, 79(4), 527-539.
9. Keynes, J. M. (1936). The General Theory of Employment, Interest and Money. London: Macmillan.
10. Duchin, F., & Lange, G. M. (1994). The Future of the Environment: Ecological Economics and Technological Change. Oxford University Press: New York.
11. SNA: Commission of the European Communities, & Inter-Secretariat Working Group on National Accounts. (1993). System of National Accounts 1993 (Vol. 2). Washington DC: International Monetary Fund.
12. Duan, Y., Dietzenbacher, E., Jiang, X., Chen, X., & Yang, C. (2018). Why has China’s vertical specialization declined?. Economic Systems Research, 30(2), 1-23.
13. De Koning, A., Huppes, G., Deetman, S., & Tukker, A. (2016). Scenarios for a 2°C world: a trade-linked input–output model with high sector detail. Climate Policy, 16(3), 301-317.
14. Duarte, R., Feng, K., Hubacek, K., Sánchez-Chóliz, J., Sarasa, C., & Sun, L. (2016). Modeling the carbon consequences of pro-environmental consumer behavior. Applied Energy, 184, 1207-1216.
15. Wiebe, K. S. (2016). The impact of renewable energy diffusion on European consumption-based emissions. Economic Systems Research, 28(2), 133-150.
16. Dejuán, Ó., López, L. A., Tobarra, M. Á., & Zafrilla, J. (2013). A post-Keynesian AGE model to forecast energy demand in Spain. Economic Systems Research, 25(3), 321-340
17. IPCC (2013). Climate change 2013: The physical science basis. Contribution of working group I to the fifth assessment report of the intergovernmental panel on climate change. Cambridge, United Kingdom: Cambridge University Press.
18. Stadler, K., Wood, R., Bulavskaya, T., Södersten, C. J., Simas, M., Schmidt, S., Usubiaga, A., Acosta-Fernández, J., Kuenen, J., Bruckner, M., Giljum, S., Lutter, S., Merciai, S., Schmidt, J. H., Theurl, M. C., Plutzar, C., Kastner, T., Eisenmenger, N., Erb, K.-H., de Koning, A. & Tukker, A. (2018). EXIOBASE 3: Developing a time series of detailed environmentally extended multi-regional input-output tables. Journal of Industrial Ecology, 22(3), 502-515.
19. Koopman, R., Wang, Z., & Wei, S. J. (2012). Estimating domestic content in exports when processing trade is pervasive. Journal of Development Economics, 99(1), 178-189.
20. Kee, H. L., & Tang, H. (2016). Domestic value added in exports: Theory and firm evidence from China. American Economic Review, 106 (6), 1402-1436.
21. Chen, Q. (2015). The Role of Household Consumption in the Chinese Economy: Input-Output Analyses. [Groningen]: University of Groningen, SOM research school.
22. Dietzenbacher, E., Los, B., Stehrer, R., Timmer, M., & De Vries, G. (2013). The construction of world input–output tables in the WIOD project. Economic Systems Research, 25(1), 71-98.
23. Timmer, M. P., Los, B., Stehrer, R., & de Vries, G. J. (2016). An anatomy of the global trade slowdown based on the WIOD 2016 release (No. GD-162). Groningen Growth and Development Centre, University of Groningen.

1. Another type of functional form that uses the logarithm of the expenditure share as the dependent variable. This approach causes problems if many expenditure shares are equal to or close to zero, like in our case with 200 products. Meanwhile, the recent development of Engel functions that includes the family characteristics (such as whether there is a child in the family and ages of family members) as independent variables [5] is also not considered, because we only have observations at the country level. [↑](#footnote-ref-1)
2. Population change is taken as an exogenous variable and the population predictions for each country during the projection period 2012-2030 are obtained from the United Nations Population Division. [↑](#footnote-ref-2)
3. Based on data from Eurostat, we checked the disposable-income-to-GDP ratio for European countries. The ratio fluctuated over the years, witnessed a decrease around 2008 and a peak in 2009, and remained relatively stable after 2011, for most countries. A similar trend is found for China: the average of the ratios from 1995 to 2011 (0.60) is close to that from 2012 to 2018 (0.58). [↑](#footnote-ref-3)
4. From 1995 to 2011, the elements of $\mathbf{w}_{1(t)}^{s}$ can be obtained from the world input-output table as $w_{1(i,t)}^{s}=\sum_{r} f_{1(i,t)}^{rs}/\sum_{i} \sum_{r} f_{1(i,t)}^{rs}$. [↑](#footnote-ref-4)
5. The historical data (from 1995 to 2010) are inflated to year 2011 using CPI data from World Bank. For the reasons for choosing the specific form of Engel function to estimate the Engel curves, see Appendix A. [↑](#footnote-ref-5)
6. The Global Warming Potential with a time horizon of 100 years for CH4, N2O, and SF6 are 34, 298, and 26087, respectively. All values are with inclusion of climate-carbon feedback (IPCC 2013, p. 714) [17]. [↑](#footnote-ref-6)
7. Dejuán et al. (2013) project the energy use at the industry level and assume that the energy coefficient in each industry evolves on the basis of the on historical trend [16]. They computed the annual rate of change of technical coefficients for each energy source (including primary energy, coal, petroleum, and gas; and secondary energy, electricity) in each industry over 2000 to 2005, by comparing the technical coefficients in the input-output table of 2005 with those in 2000 (at 2005 prices). Next, the coefficients from each energy source were extrapolated to the period 2009-2012. [↑](#footnote-ref-7)
8. Note that only real trends are extrapolated. For products whose emission coefficient is stable or does not present a significant pattern, the ‘change factors’ are set zero. The emission coefficients are deflated first before calculating the annual change rates. The product-wise deflators are obtained from [18]. [↑](#footnote-ref-8)
9. A more general setting is that households re-spend the expenditure savings on other products (on which savings are not possible), irrespective of the categories. However, since the emission multipliers of products in Shelter are generally higher than those in other categories, if savings in other categories are then re-spent in Shelter emissions will increase. The simulation results indicates that this is what actually happens. Therefore, the general setting is not in line with the idea of a low-carbon lifestyle and we choose the current setting. [↑](#footnote-ref-9)
10. This is confirmed by the results using data from the 2016 release of the World Input-Output Database [22 23], which includes updates of the input-output tables until 2014. [↑](#footnote-ref-10)
